# Prognostic impacts of diabetes status and lipoprotein(a) levels in patients with ST-segment elevation myocardial infarction: a prospective cohort study

**DOI:** 10.1186/s12933-023-01881-w

**Published:** 2023-06-26

**Authors:** Nan Li, Jinying Zhou, Runzhen Chen, Xiaoxiao Zhao, Jiannan Li, Peng Zhou, Chen Liu, Yi Chen, Ying Wang, Li Song, Shaodi Yan, Hanjun Zhao, Hongbing Yan

**Affiliations:** 1 Department of Cardiology, Fuwai Hospital, National Center for Cardiovascular Diseases, Peking Union Medical College & Chinese Academy of Medical Sciences, Beijing, China; 2 Department of Cardiology, Fuwai Hospital, Chinese Academy of Medical Sciences, Shenzhen, Shenzhen, China

**Keywords:** ST-segment elevation myocardial infarction, Lipoprotein(a), Diabetes mellitus, Prognosis

## Abstract

**Objects:**

This study aimed to investigate the impact of lipoprotein(a) [Lp(a)] levels on the prognosis of Chinese patients with ST-segment elevation myocardial infarction (STEMI), and to explore if the impact may differ in the diabetes mellitus (DM) and nonDM groups.

**Methods:**

Between March 2017 and January 2020, 1543 patients with STEMI who underwent emergency percutaneous coronary intervention (PCI) were prospectively recruited. The primary outcome was a composite of all-cause death, MI recurrence (reMI), and stroke, known as major adverse cardiovascular events (MACE). Analyses involving the Kaplan–Meier curve, Cox regression, and restricted cubic spline (RCS) were conducted.

**Results:**

During the 1446-day follow-up period, 275 patients (17.8%) experienced MACEs, including 141 with DM (20.8%) and 134 (15.5%) without DM. As for the DM group, patients with Lp(a) ≥ 50 mg/dL showed an apparently higher MACE risk compared to those with Lp(a) < 10 mg/dL (adjusted hazard ratio [HR]: 1.85, 95% confidence interval [CI]:1.10–3.11, *P* = 0.021). The RCS curve indicates that the HR for MACE appeared to increase linearly with Lp(a) levels exceeding 16.9 mg/dL. However, no similar associations were obtained in the nonDM group, with an adjusted HR value of 0.57 (Lp(a) ≥ 50 mg/dL vs. < 10 mg/dL: 95% CI 0.32–1.05, *P* = 0.071). Besides, compared to patients without DM and Lp(a) ≥ 30 mg/dL, the MACE risk of patients in the other three groups (nonDM with Lp(a) < 30 mg/dL, DM with Lp(a) < 30 mg/dL, and DM with Lp(a) ≥ 30 mg/dL) increased to 1.67-fold (95% CI 1.11–2.50, *P* = 0.013), 1.53-fold (95% CI 1.02–2.31, *P* = 0.041), and 2.08-fold (95% CI 1.33–3.26, *P* = 0.001), respectively.

**Conclusions:**

In this contemporary STEMI population, high Lp(a) levels were linked to an increased MACE risk, and very high Lp(a) levels (≥ 50 mg/dL) significantly indicated poor outcomes in patients with DM, while not for those without DM.

*Trial registration*: clinicaltrials.gov NCT: 03593928

**Supplementary Information:**

The online version contains supplementary material available at 10.1186/s12933-023-01881-w.

## Background

Lipoprotein(a) [Lp(a)] is a low-density lipoprotein-like particle comprising triglycerides, cholesteryl esters, and an apolipoprotein B-100 moiety bounded to apolipoprotein(a) [1, 2]. Accumulating evidence has manifested a positive relationship between Lp(a) levels and incident atherosclerotic cardiovascular diseases [3]. Prospective data have observed that elevated Lp(a) levels are associated with poor outcomes in patients with established cardiovascular diseases and those who underwent percutaneous coronary intervention (PCI) [4, 5]. The potential role of Lp(a) in risk stratification and the modification of residual risk is also under investigation [3].

It is acknowledged that cardiovascular risk increases apparently in diabetes mellitus (DM). However, the relationship between Lp(a) levels and DM remains unestablished [6]. Previous studies reported that patients with DM had relatively lower Lp(a) levels, and that lower Lp(a) levels were associated with higher diabetic risk [6–8]. Current studies have explored the involvement of Lp(a) level in accelerating cardiovascular risk among patients with and without diabetes; however, there was some heterogeneity among them. Konishi et al*.* reported that raised Lp(a) levels were relevant to the incidence of advanced cardiac events after PCI in patients with DM [9]. Studies by Zhang et al*.* and Jin et al*.* demonstrated that Lp(a) level was an indicator for major adverse cardiovascular events (MACE) in patients with stable coronary artery disease and pre-DM or DM [10, 11]. In contrast, a retrospective study by Silverio et al*.* observed that extremely high Lp(a) levels (> 70 mg/dL) implied an increased incidence of cardiovascular events following myocardial infarction (MI) in nondiabetic patients, but not in those with diabetes [12].

Given the inconsistent results of these studies, this study aimed to investigate the interactive effect of Lp(a) levels and diabetes status on the prognosis of patients with ST-segment elevation myocardial infarction (STEMI) who underwent primary PCI in the emergency department.

## Methods

### Population

This prospective cohort study was conducted at Fuwai Hospital (Beijing, China) and consecutively enrolled patients (age ≥ 18 years) with acute MI from March 2017 to January 2020. The exclusion criteria were as follows: (1) patients who did not undergo angiography or PCI due to extremely severe conditions, such as cardiogenic shock or complex coronary lesions; (2) not meeting the diagnosis criteria of STEMI; (3) missing Lp(a) or glycated hemoglobin A1c (HbA1c) measurements; and (4) missing follow-up information. Additional file 1: Fig. S1 shows the flowchart of this study. The diagnosis of acute MI and STEMI was based on the Fourth Universal Definition of Myocardial Infarction and up-to-date guidelines [13, 14]. DM was defined as a medical history of DM, current use of hypoglycemic drugs, or HbA1c of 6.5% or more at admission. All included patients were prescribed optimal medical therapy according to established guidelines, including antiplatelet agents, statins, beta-blockers, and renin-angiotensin system blockers [13]. The study protocol was in accordance with the Declaration of Helsinki and authorized by the Ethics Committee of Fuwai Hospital (No. 2017-866). All the patients provided informed consent upon admission.

### Information collection, blood collection, and laboratory tests

We collected patients’ information at admission, including demographics, medical history, signs and symptoms, laboratory test results, echocardiographic data, and medications at admission and discharge.

Blood samples for the HbA1c and Lp(a) tests were collected from the cubital vein after 12-h fasting (approximately seven o’clock on the following day after patients underwent PCI). Blood tests were routinely performed at the hospital’s central laboratory. High-performance liquid chromatography (Tosoh G8 Analyzer; Tosoh Bioscience, Tokyo, Japan) and immunoturbidimetry (LASAY Lp(a) Auto; SHIMA Laboratories Co., Ltd, Tokyo, Japan) were used to detect HbA1c and Lp(a) concentrations, respectively.

### Outcomes and follow up

The study outcomes were defined as follows: (1) MACE, a composite of all-cause death, recurrence of MI (reMI), and stroke, was the primary outcome of this study; (2) the secondary outcomes included individual outcomes of MACE, cardiac death, heart failure (HF) hospitalization, and unplanned revascularization. ReMI was defined as recurrent elevated troponin I levels (except for the myocardial injury caused by PCI or coronary artery bypass graft) and ischemic evidence during follow-up. Stroke was diagnosed based on focal loss of neurologic function and supported by imaging examinations. Cardiac death was defined as death caused by acute coronary syndrome, valvular heart disease, cardiomyopathy, malignant arrhythmia, or cardiac arrest. HF was identified according to guidelines and statements based on typical symptoms and signs, laboratory tests, echocardiogram, and X-ray findings [15]. Unplanned revascularization was defined as any unexpected coronary revascularization (PCI or coronary artery graft bypass) during the follow-up period.

We gathered follow-up information through telephone interviews and outpatient visits at 1, 6, and 12 months after discharge and subsequently once a year. The clinical events were identified using inpatient and outpatient records. The follow-up period was started on the day of the PCI.

### Statistical analysis

Mean ± standard deviation (SD) or median with interquartile range (IQR), and numbers (percentage) were used to summarize continuous variables and categorical variables, respectively. Differences were compared using appropriate methods based on the characteristics of variables and the number of groups. Additionally, we analyzed the relationship between blood glucose and Lp(a) levels using Spearman’s correlation coefficient and plotted with fitted linear regression curves for patients with and without DM after removing outliers of Lp(a) or glucose. Additional file 2: Fig. S2 shows the outliers in the DM and nonDM groups.

When evaluating the associations between Lp(a) levels and prognosis, we categorized patients into two groups by the normal reference limit of 30 mg/dL, which was recommended by an expert statement for the Chinese population [16], and further classified them into four groups based on detailed Lp(a) levels range values (< 10, ≥ 10–30, ≥ 30–50, and ≥ 50 mg/dL). First, the event-free survival rates of the groups were evaluated using the Kaplan–Meier curve and log-rank test. Second, the hazard ratios (HRs) and 95% confidence intervals (CIs) were computed using univariable and multivariable Cox regression analyses. The multivariable model adjusted variables as follows (*P* < 0.05 in the univariable model): age, sex, body mass index (BMI), hypertension, dyslipidemia, peripheral artery disease, chronic kidney disease (CKD), previous history of MI and PCI, Killip class, the Global Registry of Acute Coronary Events (GRACE) risk score, multiple vessels disease, estimated glomerular filtration rate, left ventricular ejection fraction, and levels of total cholesterol, low-density lipoprotein cholesterol and high-sensitivity C-reactive protein (hsCRP), as well as the baseline and peak value of cardiac troponin I (cTnI) and N-terminal pro-B-type natriuretic peptide (NT-proBNP). Finally, restricted cubic spline (RCS) analyses were used to characterize the dose–response association and explore the potential nonlinear relationships of Lp(a) levels with outcomes adjusting for the aforementioned confounders. The above analyses were conducted for overall, nonDM, and DM patients. Meanwhile, we calculated the interaction of diabetes status with the prognostic value of Lp(a) in the multivariable Cox regression model. We also compared the difference in MACE risk among groups based on Lp(a) levels and diabetes status (nonDM with Lp(a) < 30 mg/dL, nonDM with Lp(a) ≥ 30 mg/dL, DM with Lp(a) < 30 mg/dL, and DM with Lp(a) ≥ 30 mg/dL) using the Kaplan–Meier curve, log-rank test, and Cox regression analyses. Moreover, we performed additional analyses after excluding patients who suffered from PCI-related complications or had MACE within 14 days of PCI to avoid their impacts on these results.

Data analyses were conducted using SPSS software (version 26.0; IBM Corp., Armonk, New York, USA) and R (http://www.r-project.org/) statistical packages. Statistical significance was set at *P* < 0.05.

## Results

### Baseline characteristics

**Fig. 1:**
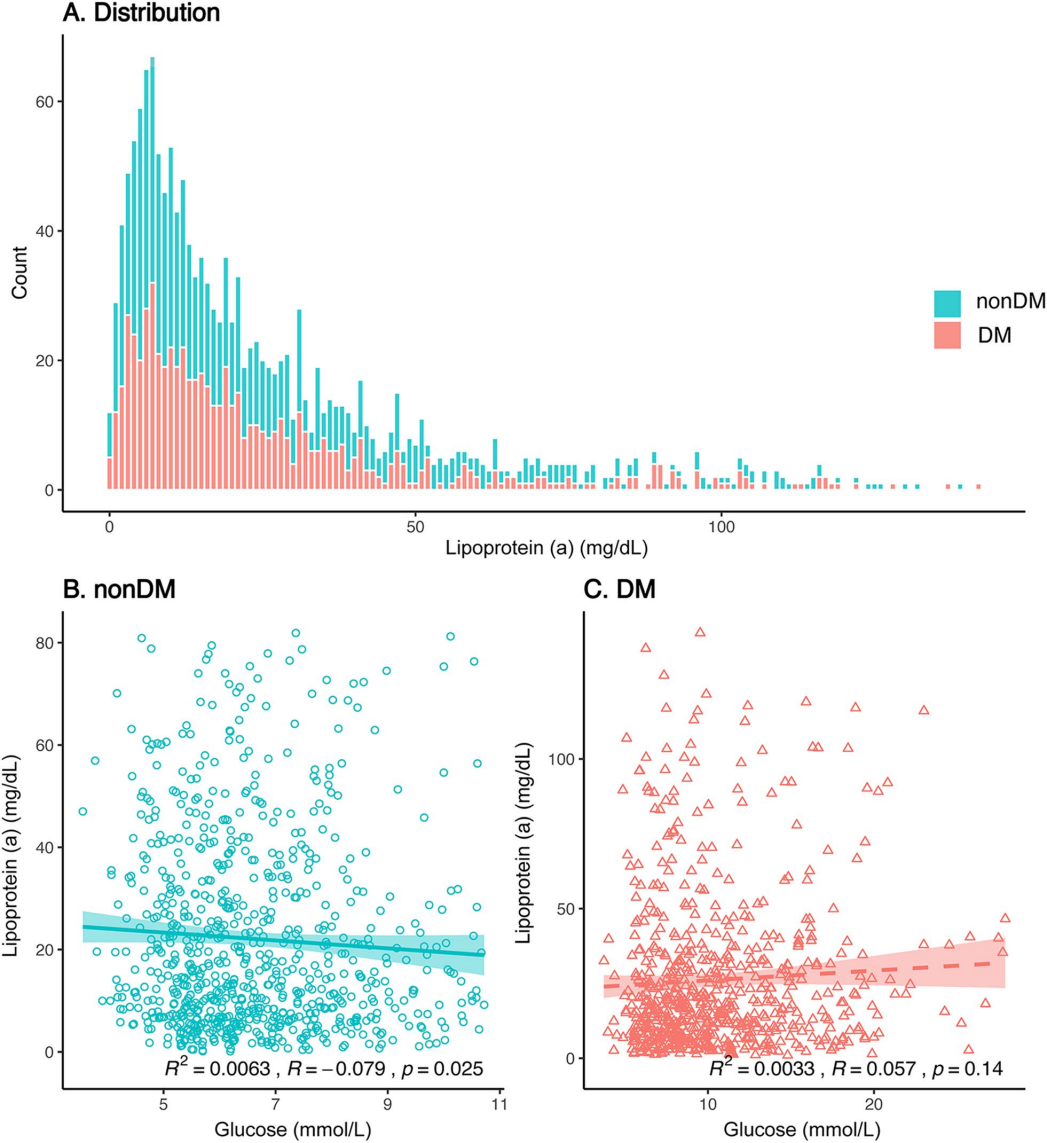
The distributions of lipoprotein(a) (**A**) and the scatter plot for the relationship between lipoprotein(a) and glucose in patients with and without diabetes mellitus (**B**, **C**). The relationships were analyzed using Spearman’s correlation coefficient and fitting linear regression curves after removing the outliers

Among the 1543 patients included in the final analysis, Lp(a) levels were elevated (≥ 30 mg/dL) in 472 patients (30.6%), and 678 (43.9%) had DM. The distribution of Lp(a) in patients with and without DM is shown in Fig. 1A. The median levels of Lp(a) were 17.3 (IQR 7.6–37.5) mg/dL in the nonDM group and 16.9 (IQR 7.8–32.8) mg/dL in the DM group (*P* = 0.586, Additional file 15: Table S1). Table 1 lists the baseline characteristics and medications of the groups according to Lp(a) levels and diabetes status. In comparison, patients with DM and elevated Lp(a) levels tended to be older, and had higher proportions of CKD, previous MI and PCI, as well as higher GRACE score, cTnI, and NT-proBNP levels. Figure 1B exhibits a weak negative correlation between Lp(a) and glucose levels in patients without DM (*R* = − 0.079, *P* = 0.025), whereas no similar observation in those with DM (Fig. 1C).

**Table 1:** Baseline characteristics according to lipoprotein(a) levels and diabetes status

	NonDM				DM			
	Total (n = 865)	Lp(a) < 30 mg/dL (n = 588)	Lp(a) ≥ 30 mg/dL (n = 277)	*P*-value	Total (n = 678)	Lp(a) < 30 mg/dL (n = 483)	Lp(a) ≥ 30 mg/dL (n = 195)	*P*-value
Age (years)	59.0 (50.5, 68.0)	59.0 (50.0, 68.0)	59.0 (51.0, 67.0)	0.855	63.0 (54.0, 69.6)	62.0 (54.0, 69.0)	64.0 (56.0, 72.0)	0.015
≥ 65 years	210 (40.1)	154 (39.1)	56 (43.1)	0.421	160 (29.3)	131 (30.3)	29 (25.7)	0.340
Female	137 (15.8)	83 (14.1)	54 (19.5)	0.043	158 (23.3)	105 (21.7)	53 (27.2)	0.129
BMI (kg/m^2^)	25.7 (23.4, 27.8)	25.8 (23.7, 28.0)	25.4 (22.9, 27.7)	0.200	25.7 (23.4, 28.0)	25.8 (23.4, 28.0)	25.5 (23.3, 27.7)	0.274
Current smoker	638 (73.8)	448 (76.2)	190 (68.6)	0.018	453 (66.8)	326 (67.5)	127 (65.1)	0.554
Hypertension	530 (61.3)	366 (62.2)	164 (59.2)	0.392	467 (68.9)	324 (67.1)	143 (73.3)	0.111
Dyslipidemia	760 (87.9)	517 (87.9)	243 (87.7)	0.933	627 (92.5)	450 (93.2)	177 (90.8)	0.284
Previous stroke	94 (10.9)	69 (11.7)	25 (9.0)	0.232	123 (18.1)	81 (16.8)	42 (21.5)	0.154
CKD	58 (6.7)	38 (6.5)	20 (7.2)	0.678	51 (7.5)	30 (6.2)	21 (10.8)	0.042
PAD	43 (5.0)	29 (4.9)	14 (5.1)	0.939	39 (5.8)	25 (5.2)	14 (7.2)	0.310
Previous MI	127 (14.7)	75 (12.8)	52 (18.8)	0.020	133 (19.6)	85 (17.6)	48 (24.6)	0.037
Previous PCI	121 (14.0)	73 (12.4)	48 (17.3)	0.052	142 (20.9)	89 (18.4)	53 (27.2)	0.011
GRACE score	105.0 (85.0, 124.0)	105.0 (84.0, 124.0)	105.0 (87.0, 123.0)	0.487	113.0 (94.0, 131.0)	110.0 (92.0, 131.0)	118.0 (102.0, 134.5)	0.004
Killip (II- IV)	98 (11.3)	68 (11.6)	30 (10.8)	0.751	111 (16.4)	81 (16.8)	30 (15.4)	0.659
LVEF (%)	55.0 (50.0, 59.0)	55.0 (50.0, 60.0)	55.0 (50.0, 58.0)	0.140	55.0 (48.0, 58.0)	55.0 (48.0, 59.0)	55.0 (49.5, 58.0)	0.892
LVEF < 50%	197 (22.8)	128 (21.8)	69 (24.9)	0.304	186 (27.4)	137 (28.4)	49 (25.1)	0.393
MVD	620 (71.7)	420 (71.4)	200 (72.2)	0.814	530 (78.2)	375 (77.6)	155 (79.5)	0.598
eGFR(ml/min/1.732 m^2^*)	89.1 (74.5, 105.9)	89.7 (75.0, 106.3)	87.7 (74.2, 105.3)	0.597	86.3 (70.7, 102.7)	88.0 (72.5, 104.9)	82.1 (67.0, 95.7)	< 0.001
Base cTnI (ng/mL)	1.0 (0.1, 5.2)	0.9 (0.1, 4.9)	1.1 (0.1, 5.9)	0.324	1.1 (0.1, 6.2)	1.1 (0.1, 5.9)	1.3 (0.2, 7.9)	0.354
Peak cTnI (ng/mL)	16.0 (5.8, 37.3)	15.5 (5.6, 37.3)	16.6 (6.8, 37.3)	0.495	16.1 (4.2, 38.5)	14.5 (4.0, 34.9)	21.3 (4.9, 48.8)	0.025
Base NT-proBNP (pg/mL)	229.1 (58.8, 802.2)	213.2 (54.1, 751.5)	264.2 (68.0, 899.2)	0.079	344.6 (84.8, 1104.0)	293.7 (75.8, 1024.0)	523.2 (124.0, 1292.0)	0.002
Peak NT-proBNP (pg/mL)	1385.0 (536.6, 3113.0)	1284.0 (537.7, 3034.0)	1629.0 (532.6, 3213.0)	0.168	1622.0 (673.4, 3508.0)	1429.0 (584.0, 3184.0)	2078.0 (920.0, 4460.0)	0.004
TC (mmol/L)	4.3 (3.6, 5.0)	4.2 (3.5, 4.9)	4.5 (3.8, 5.2)	< 0.001	4.2 (3.6, 5.0)	4.2 (3.6, 4.9)	4.2 (3.5, 5.1)	0.713
Triglyceride (mmol/L)	1.4 (0.9, 1.9)	1.4 (0.9, 2.0)	1.3 (1.0, 1.9)	0.689	1.5 (1.1, 2.2)	1.6 (1.1, 2.3)	1.5 (1.1, 2.1)	0.335
LDL-C (mmol/L)	2.7 (2.1, 3.3)	2.6 (2.0, 3.2)	2.9 (2.2, 3.4)	< 0.001	2.6 (2.0, 3.2)	2.6 (2.0, 3.2)	2.6 (2.0, 3.3)	0.453
HDL-C (mmol/L)	1.1 (0.9, 1.3)	1.1 (0.9, 1.2)	1.1 (0.9, 1.3)	0.190	1.0 (0.9, 1.2)	1.0 (0.9, 1.2)	1.0 (0.9, 1.2)	0.657
Lipoprotein(a) (mg/dL)	17.3 (7.6, 37.5)	10.4 (5.7, 17.9)	50.5 (38.8, 73.0)	< 0.001	16.9 (7.8, 32.8)	11.5 (6.2, 18.6)	47.6 (36.5, 77.0)	< 0.001
Glucose (mmol/L)	6.4 (5.5, 7.6)	6.4 (5.5, 7.8)	6.4 (5.5, 7.4)	0.173	9.6 (7.4, 12.8)	9.4 (7.3, 12.5)	9.7 (7.5, 13.5)	0.217
HbA1c (%)	5.7 (5.5, 6.0)	5.7 (5.5, 6.0)	5.7 (5.5, 6.0)	0.489	7.6 (6.8, 8.9)	7.5 (6.8, 8.8)	7.8 (6.8, 9.2)	0.133
hsCRP (mg/L)	5.5 (2.0, 10.6)	4.7 (1.8, 10.4)	7.5 (2.5, 10.9)	0.004	6.6 (2.5, 11.1)	6.3 (2.5, 11.1)	6.8 (2.5, 11.4)	0.285
Medication								
Baseline statins	184 (21.3)	127 (21.6)	57 (20.6)	0.732	171 (25.2)	110 (22.8)	61 (31.3)	0.021
Follow-up statins	829 (96.4)	557 (95.5)	272 (98.2)	0.051	640 (95.7)	453 (95.0)	187 (97.4)	0.163
Aspirin	828 (96.3)	560 (96.1)	268 (96.8)	0.614	646 (96.6)	459 (96.2)	187 (97.4)	0.453
Ticagrelor	438 (50.6)	308 (52.8)	130 (46.9)	0.106	311 (46.5)	229 (48.0)	82 (42.7)	0.214
Clopidogrel	424 (49.3)	278 (47.7)	146 (52.7)	0.169	353 (52.8)	245 (51.4)	108 (56.2)	0.252
ACEI/ARB	623 (72.4)	431 (73.9)	192 (69.3)	0.157	481 (71.9)	345 (72.3)	136 (70.8)	0.697
Βeta blocker	739 (85.9)	497 (85.2)	242 (87.4)	0.404	591 (88.3)	419 (87.8)	172 (89.6)	0.525
Insulin	-				155 (22.9)	103 (21.3)	52 (26.7)	0.134

Continuous variables are presented as medians (25–75th percentiles), and categorical variables are reported as counts (%). ACEI/ARB indicates angiotensin-converting enzyme inhibitors/angiotensin receptor blockers; BMI, body mass index; CKD, chronic kidney disease; cTnI, cardiac troponin I; DM, diabetes mellitus; GRACE, the Global Registry of Acute Coronary Events; HbA1c, hemoglobin A1c; HDL-C, high-density lipoprotein cholesterol; hsCRP, high-sensitivity C-reactive protein; LDL-C, low-density lipoprotein cholesterol; LVEF, left ventricular ejection fraction; MVD, multiple vessels disease; NT-proBNP, N-terminal pro B-type natriuretic peptide; PAD, peripheral artery disease; PCI, percutaneous coronary intervention; TC, total cholesterol. *estimated glomerular filtration rate (eGFR) was calculated according to the Modification of Diet in Renal Disease formula.

### Long-term outcomes in overall patients

**Fig. 2:**
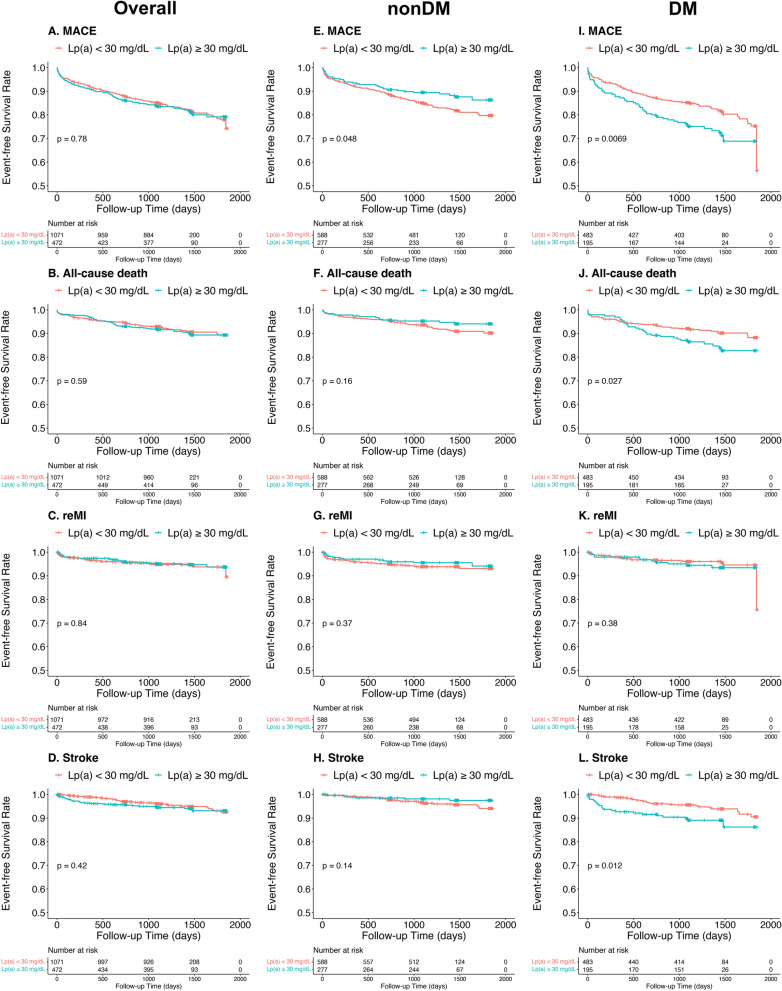
Kaplan–Meier curves for cumulative event-free survival rate between groups by lipoprotein(a) levels in overall, nonDM, and DM patients. DM, diabetes mellitus; MACE, major adverse cardiovascular event (a composite of all-cause death, recurrent myocardial infarction, and stroke); reMI, recurrent myocardial infarction

**Fig. 3:**
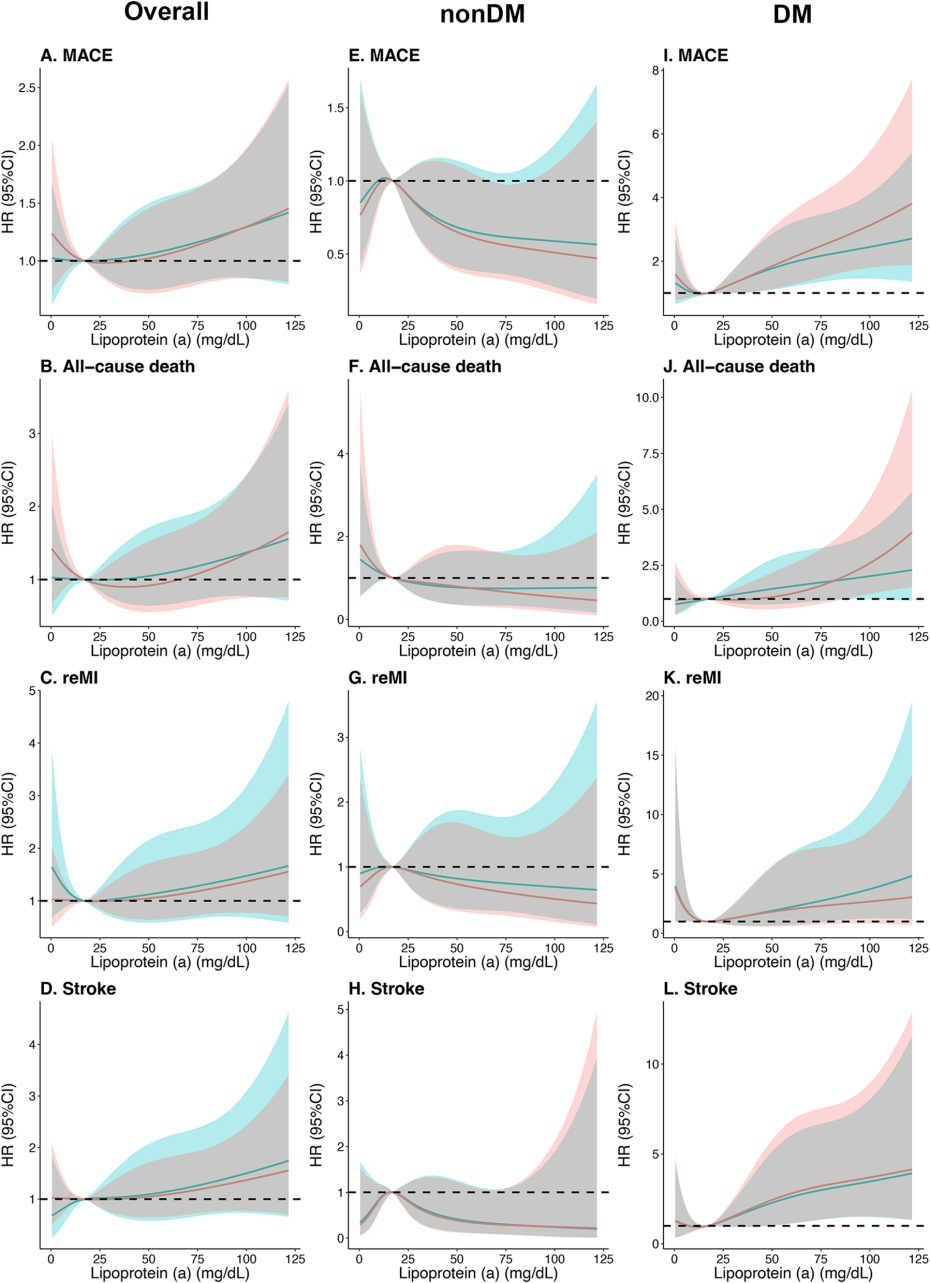
Continuous hazard ratio across lipoprotein(a) levels for major adverse cardiovascular events in overall, nonDM, and DM patients. DM, diabetes mellitus; HR, hazard ratio; MACE, major adverse cardiovascular event (a composite of all-cause death, recurrent myocardial infarction, and stroke); reMI, recurrent myocardial infarction. The blue line indicates unadjusted fits with the 95% confidence intervals shown as the blue-shaded area; the red line indicates adjusted fits with the 95% confidence intervals shown as the red-shaded area (adjusted for age, sex, body mass index, hypertension, dyslipidemia, peripheral artery disease, chronic kidney disease, previous history of myocardial infarction and percutaneous coronary intervention, Killip class, the Global Registry of Acute Coronary Events risk score, multiple vessels disease, estimated glomerular filtration rate, left ventricular ejection fraction, and levels of total cholesterol, low-density lipoprotein cholesterol and high-sensitivity C-reactive protein, as well as the baseline and peak value of cardiac troponin I and N-terminal pro-B-type natriuretic peptide)

Over a follow-up time of 1446 (IQR 1091–1472) days, 275 patients (17.8%) experienced MACEs, consisting of 141 with DM (20.8%) and 134 (15.5%) without DM. The Kaplan–Meier curves presented that the event-free survival rates among the groups based on Lp(a) levels in overall patients were not statistically different (all *P*_*log-rank*_ > 0.05, Fig. 2 and Additional file 3: Fig. S3, Additional file 4: Fig. S4, Additional file 5: Fig. S5). The Cox regression analysis (Table 2 and Additional file 16: Table S2) and the RCS curves (Fig. 3, Additional file 6: Fig. S6, and Additional file 17: Table S3) indicated no significant correlations between the risks of outcomes and Lp(a) levels except that patients with 10 ≤ Lp(a) < 30 mg/dL had a higher risk of unplanned revascularization compared to those with Lp(a) < 10 mg/dL (adjusted HR 1.37, 95%CI 1.01–1.86, *P* = 0.042; Additional file 16: Table S2). Furthermore, we analyzed the interaction between diabetes status and Lp(a) levels in terms of prognosis in the multivariable Cox regression model. *P-*values for the interaction of diabetes status on the associations between MACE and Lp(a) cutoff (30 mg/dL), Lp(a) per SD, and groups (10, 30, and 50 mg/dL) were 0.002, 0.001, and 0.007, respectively. Then, the associations between Lp(a) levels and MACE risk were analyzed in patients with and without DM, respectively.

**Table 2:** Association between MACE and lipoprotein(a) levels

Lp(a) (mg/dL)	Event (n/%)	Crude HR (95%CI)	*P*-value	Adjusted HR (95%CI)*	*P*-value
Overall patients^†^					
Lp(a) ≥ 30 vs < 30	86 (18.2)	1.04 (0.80–1.34)	0.784	0.91 (0.70–1.18)	0.461
Lp(a) per SD	275 (17.8)	1.07 (0.95–1.19)	0.260	1.03 (0.92–1.16)	0.627
Lp(a) < 10	87 (17.6)	1 (Ref)		1 (Ref)	
10 ≤ Lp(a) < 30	102 (17.7)	1.03 (0.77–1.37)	0.863	0.94 (0.70–1.25)	0.660
30 ≤ Lp(a) < 50	44 (18.4)	1.08 (0.75–1.55)	0.686	0.86 (0.59–1.25)	0.429
Lp(a) ≥ 50	42 (18.0)	1.02 (0.71–1.48)	0.903	0.89 (0.61–1.29)	0.532
Patients without DM					
Lp(a) ≥ 30 vs < 30	33 (11.9)	0.67 (0.45–1.00)	0.049	0.63 (0.42–0.95)	0.029
Lp(a) per SD	134 (15.5)	0.85 (0.71–1.03)	0.099	0.84 (0.69–1.01)	0.070
Lp(a) < 10	48 (17.0)	1 (Ref)		1 (Ref)	
10 ≤ Lp(a) < 30	53 (17.3)	1.05 (0.71–1.55)	0.806	1.13 (0.75–1.70)	0.553
30 ≤ Lp(a) < 50	18 (13.2)	0.77 (0.45–1.33)	0.347	0.79 (0.45–1.39)	0.418
Lp(a) ≥ 50	15 (10.6)	0.62 (0.34–1.10)	0.101	0.57 (0.32–1.05)	0.071
Patients with DM					
Lp(a) ≥ 30 vs < 30	53 (27.2)	1.59 (1.13–2.24)	0.007	1.43 (1.00–2.05)	0.050
Lp(a) per SD	141 (20.8)	1.28 (1.12–1.47)	< 0.001	1.33 (1.15–1.55)	< 0.001
Lp(a) < 10	39 (18.4)	1 (Ref)		1 (Ref)	
10 ≤ Lp(a) < 30	49 (18.1)	0.99 (0.65–1.51)	0.975	0.80 (0.52–1.23)	0.310
30 ≤ Lp(a) < 50	26 (25.2)	1.53 (0.93–2.51)	0.095	0.93 (0.55–1.57)	0.798
Lp(a) ≥ 50	27 (29.3)	1.65 (1.01–2.70)	0.046	1.85 (1.10–3.11)	0.021

DM, diabetes mellitus; HR, hazard ratio; Lp(a), lipoprotein (a); MACE, major adverse cardiovascular event (a composite of all-cause death, recurrent myocardial infarction, and stroke)

^*^Adjusted for age, sex, body mass index, hypertension, dyslipidemia, peripheral artery disease, chronic kidney disease, previous history of myocardial infarction and percutaneous coronary intervention, Killip class, the Global Registry of Acute Coronary Events risk score, multiple vessels disease, estimated glomerular filtration rate, left ventricular ejection fraction, and levels of total cholesterol, low-density lipoprotein cholesterol and high-sensitivity C-reactive protein, as well as the baseline and peak value of cardiac troponin I and N-terminal pro-B-type natriuretic peptide

^†^*P-*values for the interaction of diabetes status on the associations between MACE and Lp(a) cutoff (30 mg/dL), Lp(a) per SD, and groups (10, 30, and 50 mg/dL) in the multivariable Cox regression were 0.002, 0.001, and 0.007, respectively

### Long-term outcomes in patients without DM

In the nonDM group, the Kaplan–Meier curves showed a significant difference in MACE risk between patients with Lp(a) ≥ 30 and < 30 mg/dL (*P*_*log-rank*_ = 0.048, Fig. 2E), while not in the four groups based on detailed Lp(a) level ranges (*P*_*log-rank*_ = 0.23, Additional file 4: Fig. S4E). The Cox regression indicated that patients with Lp(a) ≥ 30 mg/dL had a decreased MACE risk compared to those with Lp(a) < 30 mg/dL (adjusted HR 0.63, 95%CI 0.42–0.95, *P* = 0.029, Table 2), while no similar associations with MACE risk were obtained in the four detailed groups and per 1-SD change (26.9 mg/dL) of Lp(a) levels (Table 2). The RCS curves in Fig. 3 also did not yield a significant association between Lp(a) levels and MACE risk.

Regarding the risks of the secondary outcomes (all-cause death, reMI, stroke, cardiac death, HF hospitalization, and unplanned revascularization), there were no significant differences regardless of the classification of Lp(a) in the Kaplan–Meier curves (Fig. 2 and Additional file 3: Fig. S3, Additional file 4: Fig. S4, Additional file 5: Fig. S5) and the Cox regression models (Additional file 16: Table S2). Additionally, the RCS curves for these relationships were not statistically significant (Fig. 3, Additional file 6: Fig. S6, and Additional file 17: Table S3).

### Long-term outcomes in patients with DM

In the DM group, Kaplan–Meier curves exhibited that patients with Lp(a) ≥ 30 mg/dL had elevated risk of MACE (*P*_*log-rank*_ = 0.007, Fig. 2I), whereas no significant differences were detected in the four groups based on Lp(a) levels (*P*_*log-rank*_ = 0.06, S Additional file 4: Fig. S4I). The Cox regression analysis results showed that patients with Lp(a) ≥ 50 mg/dL had a significantly higher risk of MACE compared to patients with Lp(a) < 10 mg/dL (adjusted HR 1.85, 95%CI 1.10–3.11, *P* = 0.021, Table 2). Moreover, the MACE risk increased by 33% per 1-SD change (26.3 mg/dL) in Lp(a) levels (adjusted HR 1.33, 95%CI 1.15–1.55, *P* < 0.001, Table 2). RCS curves revealed that the HR for MACE increases linearly as Lp(a) levels exceed 16.9 mg/dL (Fig. 3I).

As for secondary outcomes in patients with DM, the Kaplan–Meier curves showed significant differences between patients with Lp(a) ≥ 30 mg/dL and < 30 mg/dL in risks of all-cause death, stroke, cardiac death, and HF hospitalization (Fig. 2 and Additional file 3: Fig. S3). Kaplan–Meier analysis also showed that only the risks of cardiac death and HF hospitalization showed significant differences among the four groups (Additional file 4: Fig. S4, Additional file 5: Fig. S5). In the Cox regression, per 1-SD change in Lp(a) levels were correlated with 32%, 46%, and 68% increased risks of all-cause death, stroke, and cardiac death, respectively (adjusted HR 1.32, 95%CI 1.06–1.64, *P* = 0.012; adjusted HR 1.46, 95%CI 1.16–1.84, *P* = 0.001; and adjusted HR 1.68, 95% CI 1.29–2.20, *P* < 0.001, respectively; Additional file 16: Table S2). Patients with Lp(a) ≥ 50 mg/dL had higher risks of stroke and cardiac death compared to those with Lp(a) < 10 mg/dL (adjusted HR 2.47, 95%CI 1.07–5.70, *P* = 0.035; adjusted HR 5.60, 95%CI 1.93–16.30, *P* = 0.002; Additional file 16: Table S2). Patients with 30 ≤ Lp(a) < 50 mg/dL had a higher risk of HF hospitalization compared to those with Lp(a) < 10 mg/dL (adjusted HR 4.99, 95%CI 1.21–20.57, *P* = 0.026; Additional file 16: Table S2). Additionally, the RCS curves also displayed positive relationships between Lp(a) levels and the risks of stroke and cardiac death (Fig. 3 and Additional file 6: Fig. S6, and Additional file 17: Table S3).

### Long-term outcomes in groups based on Lp(a) levels and diabetes status

The Kaplan–Meier curves showed that DM patients with Lp(a) ≥ 30 mg/dL had the highest risks of MACE, all-cause death, stroke, and cardiac death among the four groups (*P *_*log-rank*_ < 0.05, Additional file 7: Fig. S7). In the Cox regression model, compared to nonDM patients with Lp(a) < 30 mg/dL, those with Lp(a) ≥ 30 mg/dL and DM had higher MACE risk (HR 1.71, 95%CI 1.23–2.39, *P* = 0.002, Additional file 18: Table S4), whereas the significance diminished after adjusting for the confounders (adjusted HR 1.25, 95%CI 0.88–1.77, *P* = 0.208, Additional file 18: Table S4). Compared with nonDM patients with Lp(a) ≥ 30 mg/dL, the MACE risk of the other three groups increased to 1.67-fold, 1.53-fold, and 2.08-fold, respectively (Additional file 18: Table S4). Regarding secondary outcomes, DM patients with Lp(a) ≥ 30 mg/dL showed elevated risks of all-cause death, stroke, and cardiac death compared to nonDM patients with Lp(a) ≥ 30 mg/dL (Additional file 18: Table S4).

### Impacts of PCI-related complications and short-term MACE on outcomes

A total of 11 patients (0.7%) suffered from PCI-related complications, and 17 (1.1%) experienced MACE within 14 days. After excluding these patients, the associations between risks of outcomes and Lp(a) levels were insignificant in overall patients (n = 1515), except that the risk of cardiac death increased by 25% per 1-SD change in Lp(a) levels (adjusted HR 1.25, 95%CI 1.00–1.25, *P* = 0.048, Additional file 19: Table S5). As for patients without DM (n = 853), there were no relationships between Lp(a) levels and outcomes according to the Kaplan–Meier curves, multivariable Cox regression, and RCS fits (Additional file 8: Fig. S8, Additional file 9: Fig. S9, Additional file 10: Fig. S10, Additional file 11: Fig. S11, Additional file 12: Fig. S12, Additional file 13: Fig. S13, Additional file 14: Fig. S14 and Additional file 19: Tables S5, Additional file 20: Table S6, Additional file 21: Table S7). As for patients with DM (n = 662), increasing Lp(a) levels were mainly linked to the risks of MACE, all-cause death, stroke, and cardiac death, similar to the results obtained in the previous section (Additional file 8: Fig. S8, Additional file 9: Fig. S9, Additional file 10: Fig. S10, Additional file 11: Fig. S11, Additional file 12: Fig. S12, Additional file 13: Fig. S13, Additional file 14: Fig. S14 and Additional file 19: Tables S5, Additional file 20: Table S6, Additional file 21: Table S7).

## Discussion

This study focused on a contemporary cohort of Chinese patients with STMEI who underwent emergency PCI, and explored the impact of diabetes status on the value of Lp(a) levels in long-term outcomes. The primary findings were that high Lp(a) levels were related to increased MACE risk and very high Lp(a) levels (≥ 50 mg/dL) significantly indicated poor outcomes in patients with DM, while not in those without DM. Meanwhile, we found a weak negative relationship between Lp(a) and glucose levels in the nonDM group, while not in the DM group.

Lp(a) is synthesized in the liver and cleared by the liver, kidney, or a combination of mechanisms [1, 16, 17]. Although Lp(a) level predominantly depends on genetics, other factors, such as renal and hepatic function, inflammation, and hormone levels, may also affect its level [18]. For instance, impaired renal function may increase Lp(a) levels by reducing the catabolism of large isomers [19], and impaired hepatic function may cause the production to be reduced [20]. The contribution of Lp(a) in accelerating cardiovascular disease involves several mechanisms, as follows [1, 3]: (1) promoting the formation of reactive oxygen species, which further augments endothelial permeability, produces cytokine, and results in inflammation, apoptosis, and vascular wall remodeling; (2) accelerating the uptake of oxidized low-density lipoprotein cholesterol by macrophages-induced formatting of foam cells and subsequent atherogenesis; and (3) facilitating monocyte adhesion and migration by the interaction of apolipoprotein(a) with β2-integrin Mac1.

It is well-established that the risk of all-cause and cardiovascular death in patients with DM is much higher than that in patients without DM due to macrovascular and microvascular complications [21–23]. However, the relationship between Lp(a) levels and diabetes and its potential mechanism are undergoing investigation and remain elusive. Previous studies demonstrated that lower Lp(a) levels were linked to an increased diabetes risk [6, 24]. Some have identified that Lp(a) might indicate insulin resistance and trigger systemic low-grade inflammation and enhanced autoimmune reactions [25, 26]. The results of our current study suggested a slightly inverse relationship between Lp(a) levels and blood glucose levels in patients without DM. This might provide an additional reference for this phenomenon.

Given the inverse association between Lp(a) levels and diabetic risk, current studies have examined the impact of diabetic status on Lp(a)-associated cardiovascular diseases. Konishi et al*.* demonstrated a relationship between increased Lp(a) levels and high incidences of cardiac death and acute coronary syndrome after PCI in patients with DM [9]. Zhang et al*.* and Jin et al*.* found that Lp(a) level was a risk indicator for a composite endpoint (including nonfatal MI, stroke, and cardiovascular mortality) in patients with stable coronary artery disease and pre-DM or DM [10, 11]. Likewise, our current study primarily revealed different relationship curves between Lp(a) levels and HR for MACE in patients with and without DM (Fig. 3). That was, elevated Lp(a) levels were associated with increased MACE risk in DM patients, while not for nonDM patients. For one thing, studies have shown that Lp(a) is associated with an increased risk of both micro and macrovascular complications in diabetes and revealed that elevated Lp(a) and glucose levels might have a synergistic effect, leading to enhanced damage to the vascular endothelium, greater susceptibility to vascular complications, and a worse prognosis [6, 27, 28]. In contrast, nondiabetic patients have fewer combined cardiovascular risk factors than diabetic patients, so the prognosis of nondiabetic patients may be less susceptible to weaker risk factors. These may explain why the impacts of Lp(a) on prognosis were varying in patients with and without DM. The differential prognostic value of Lp(a) also implies that Lp(a) may act differently to promote atherosclerosis in patients with and without DM, leading to its different weighting compared to other cardiovascular risk factors. Therefore, more attention should be paid to Lp(a) in clinical practice because of its complicated effect on cardiometabolic diseases. It is worth noting that Chinese patients showed an obviously increased MACE risk when Lp(a) levels were above 50 mg/dL, rather than 70 mg/dL. This may be due to the lower Lp(a) levels in the Chinese population than those in other countries and regions [16].

In addition, this study did not detect a significant relationship between Lp(a) levels and MACE risk in overall patients, which was different from previous studies [29, 30]. There are several possible reasons: (1) Male patients accounted for 80% of the patients included in this study, which was much higher than that in other studies [31]. A study by Cui et al*.* reported that a 5-year age increase was associated with a median increase of 2.03 mg/L in Lp(a) levels in males and 6.87 mg/L in females, whereas the effect of age on the median Lp(a) levels in females significantly weakened after the age of 55 to 60 years [32]. These observations imply the complex influence of sex and age on Lp(a) levels. Therefore, the sex composition of this study might be one of the possible reasons for the different results. (2) Compared to patients with stable coronary artery disease, those with STEMI have more cardiovascular risk factors and a higher inflammatory burden, which may result in the impact of Lp(a) on outcomes interacting with other risk factors. For example, our previous study found that an elevated risk of MACE was seen in patients with higher levels of Lp(a) levels only in the setting of high hsCRP levels (hsCRP ≥ 2 mg/L) [5]. (3) The blood samples for Lp(a) measurement in this study were collected on the following day after patients underwent PCI for STEMI. Previous studies have revealed that Lp(a) levels may increase in the first few days after myocardial infarction [33, 34]. STEMI is a severe condition that affects the body and its response varies depending on patients’ characteristics, which might lead to differential impacts of Lp(a) on outcomes. Therefore, the prognostic impact of Lp(a) should be interpreted cautiously in the systematic context of the patient.

Regarding Lp(a)-associated cardiovascular risk, how to manage patients with high Lp(a) levels is becoming a challenge for clinicians. Evidence suggests that proprotein convertase subtilisin/kexin type 9 inhibitors could reduce Lp(a)-associated cardiovascular risk, and the benefit is likely related to the degree of Lp(a) reduction [29, 35]. Niacin, cholesteryl ester transfer protein inhibitors, and antisense oligonucleotides and small interfering RNA agents targeting apolipoprotein B and LPA, could reduce Lp(a) levels [24]. However, it remains unclear whether they could provide cardiovascular benefits [24]. Further investigation is needed to determine when to initiate Lp(a) lowering therapy and figure out to what level the reduction can lead to clinical benefits.

## Limitations

This study had several limitations. First, this study was a single-center, observational study among Chinese patients with STEMI. Therefore, when interpreting and extrapolating these results, it is essential to note the characteristics of the STEMI population and the relatively lower Lp(a) levels in the Chinese population. Second, Lp(a) levels were measured only once in this study. Since it is an acute-phase protein to some degree, repeated measurements may provide more information of the impact of Lp(a) levels on prognosis. Third, we did not measure insulin concentrations, which could have provided more information on the association among Lp(a), DM, and cardiovascular events. Finally, measuring Lp(a) levels in clinical practice is challenging. Ideally, Lp(a) should be measured in molar units to ensure that each Lp(a) particle is recognized only once. In this study, Lp(a) levels were measured using immunoturbidimetric methods, which are commonly used in clinical practice [36]. It is crucial to note that this method could be affected by variations in the Lp(a) particle size, or even more by the presence of lipid-free or fragmented apo(a). The Lp(a) consensus statement of the European Atherosclerosis Society pointed out that the assays available in clinical practice are not yet ideal, but are most likely adequate for risk discrimination [37]. Concerning this, it is urgent to standardize Lp(a) assays. Efforts regarding this standardization are also underway [38]. Therefore, further investigation is needed to be conducted with larger sample sizes, more detailed groups, and more accurate measurement methods to explore and explain the association and its potential mechanism.

## Conclusions

In this cohort of STMEI patients undergoing emergency PCI, elevated Lp(a) levels were associated with a higher MACE risk and very high Lp(a) levels (≥ 50 mg/dL) independently indicated poor outcomes in DM patients, while not for nonDM patients. It is crucial for patients with STEMI to measure Lp(a) levels and to comprehensively assess the prognostic value of Lp(a), particularly for patients with DM.

## Supplementary Information

**Additional file 1: Figure S1.** Subject disposition flow chart. AMI, acute myocardial infarction; DM, diabetes mellitus; HbA1c, hemoglobin A1c; Lp, lipoprotein; PCI, percutaneous coronary intervention; STEMI, ST-segment elevation myocardial infarction.

**Additional file 2: Figure S2.** The scatter plot for the relationship between lipoproteinand glucose in patients with and without diabetes mellitus. Outliers are tested using a box plot test and marked in red, with 69in the nonDM group and 70in the DM group.

**Additional file 3: Figure S3.** Kaplan–Meier curves for cumulative event-free survival rate between groups by lipoproteinlevels in overall, nonDM, and DM patients. DM, diabetes mellitus.

**Additional file 4: Figure S4.** Kaplan–Meier curves for cumulative event-free survival rate among groups by the detailed lipoproteinlevels in overall, nonDM, and DM patients. DM, diabetes mellitus; MACE, major adverse cardiovascular event; reMI, recurrent myocardial infarction.

**Additional file 5: Figure S5.** Kaplan–Meier curves for cumulative event-free survival rate among groups by the detailed lipoproteinlevels in overall, nonDM, and DM patients. DM, diabetes mellitus.

**Additional file 6: Figure S6.** Continuous hazard ratio across lipoproteinlevels for secondary outcomes in overall, nonDM, and DM patients. DM, diabetes mellitus; HR, hazard ratio. The blue line indicates unadjusted fits with the 95% confidence intervals shown as the blue-shaded area; the red line indicates adjusted fits with the 95% confidence intervals shown as the red-shaded area.

**Additional file 7: Figure S7.** Kaplan–Meier curves for cumulative event-free survival rate among groups based on lipoproteinlevels and diabetes status. MACE, major adverse cardiovascular event; reMI, recurrent myocardial infarction.

**Additional file 8: Figure S8.** Kaplan–Meier curves for cumulative event-free survival rate among groups by lipoproteinlevels in patients without MACEs within 14 days or PCI-related complications. DM, diabetes mellitus; MACE, major adverse cardiovascular event; reMI, recurrent myocardial infarction.

**Additional file 9: Figure S9.** Kaplan–Meier curves for cumulative event-free survival rate between groups by lipoproteinlevels in patients without MACEs within 14 days or PCI-related complications. DM, diabetes mellitus.

**Additional file 10: Figure S10.** Kaplan–Meier curves for cumulative event-free survival rate among groups by the detailed lipoproteinlevels in patients without MACEs within 14 days or PCI-related complications. DM, diabetes mellitus; MACE, major adverse cardiovascular event; reMI, recurrent myocardial infarction.

**Additional file 11: Figure S11.** Kaplan–Meier curves for cumulative event-free survival rate among groups by the detailed lipoproteinlevels in patients without MACEs within 14 days or PCI-related complications. DM, diabetes mellitus.

**Additional file 12: Figure S12.** Kaplan–Meier curves for cumulative event-free survival rate among groups based on lipoproteinlevels and diabetes status in patients without MACEs within 14 days or PCI-related complications. MACE, major adverse cardiovascular event; reMI, recurrent myocardial infarction.

**Additional file 13: Figure S13.** Continuous hazard ratio across lipoproteinlevels for the primary outcome in patients without MACEs within 14 days or PCI-related complications. DM, diabetes mellitus; HR, hazard ratio; MACE, major adverse cardiovascular event; reMI, recurrent myocardial infarction. The blue line indicates unadjusted fits with the 95% confidence intervals shown as the blue-shaded area; the red line indicates adjusted fits with the 95% confidence intervals shown as the red-shaded area.

**Additional file 14: Figure S14.** Continuous hazard ratio across lipoproteinlevels for secondary outcomes in patients without MACEs within 14 days or PCI-related complications. DM, diabetes mellitus; HR, hazard ratio. The blue line indicates unadjusted fits with the 95% confidence intervals shown as the blue-shaded area; the red line indicates adjusted fits with the 95% confidence intervals shown as the red-shaded area.

**Additional file 15: Table S1.** Baseline characteristics according to diabetes status.

**Additional file 16: Table S2.** Association between lipoproteinlevels and risks of outcomes.

**Additional file 17: Table S3.** The details of restricted cubic spline fits for the relationships between continuous lipoproteinlevels and risks of outcomes.

**Additional file 18: Table S4.** Association between risks of outcomes and groups based on lipoproteinlevels and diabetes status.

**Additional file 19: Table S5.** Association between lipoproteinlevels and risks of outcomes in patients without MACEs within 14 days or PCI-related complications.

**Additional file 20: Table S6.** Association between risks of outcomes and groups based on lipoproteinlevels and diabetes status in patients without MACEs within 14 days or PCI-related complications.

**Additional file 21: Table S7.** The details of restricted cubic spline fits for the relationships between continuous lipoproteinlevels and risks of outcomes in patients without MACEs within 14 days or PCI-related complications.

## Data Availability

Data are available upon reasonable request by contacting the corresponding author.

## Abbreviations

BMI: Body mass index
CIs: Confidence intervals
CKD: Chronic kidney disease
cTnI: Cardiac troponin I
DM: Diabetes mellitus
GRACE: Global Registry of Acute Coronary Events
HbA1c: Hemoglobin A1c
HRs: Hazard ratios
hsCRP: High-sensitivity C-reactive protein
IQR: Interquartile range
Lp(a): Lipoprotein(a)
MACE: Major adverse cardiovascular event
MI: Myocardial infarction
NT-proBNP: N-terminal pro-B-type natriuretic peptide
PCI: Percutaneous coronary intervention
RCS: Restricted cubic spline
SD: Standard deviation
STEMI: ST-segment elevation myocardial infarction
