# Supplementary figures and images for: Prognostic impacts of diabetes status and lipoprotein(a) levels in patients with ST-segment elevation myocardial infarction: a prospective cohort study
Source: Cardiovasc Diabetol. 2023 Jun 26;22:151. doi: 10.1186/s12933-023-01881-w (PMC10294355; doi:10.1186/s12933-023-01881-w)

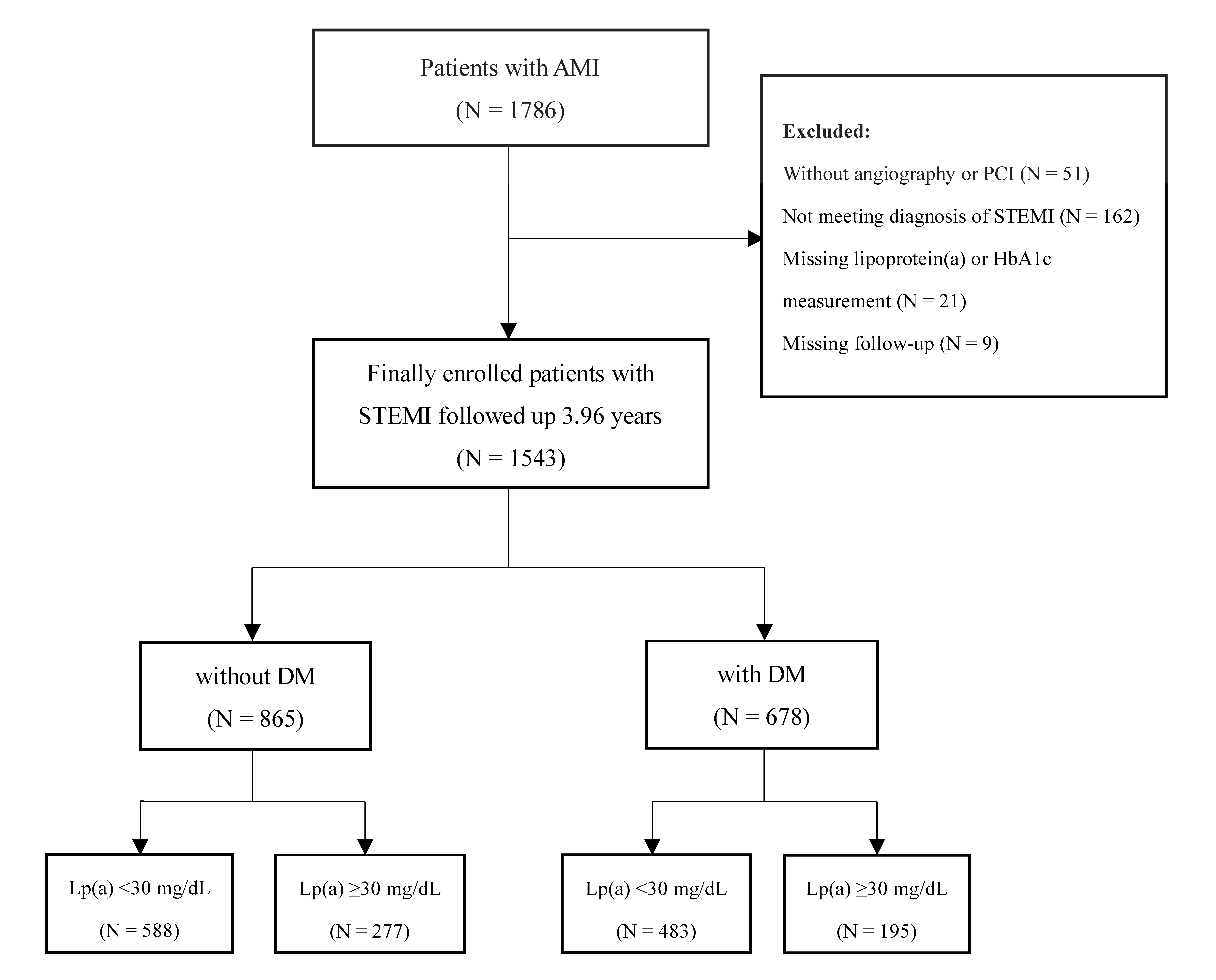

Supplement: Supplementary file 1 — Additional file 1: Figure S1. Subject disposition flow chart. AMI, acute myocardial infarction; DM, diabetes mellitus; HbA1c, hemoglobin A1c; Lp, lipoprotein; PCI, percutaneous coronary intervention; STEMI, ST-segment elevation myocardial infarction. [file 12933_2023_1881_MOESM1_ESM.tiff]

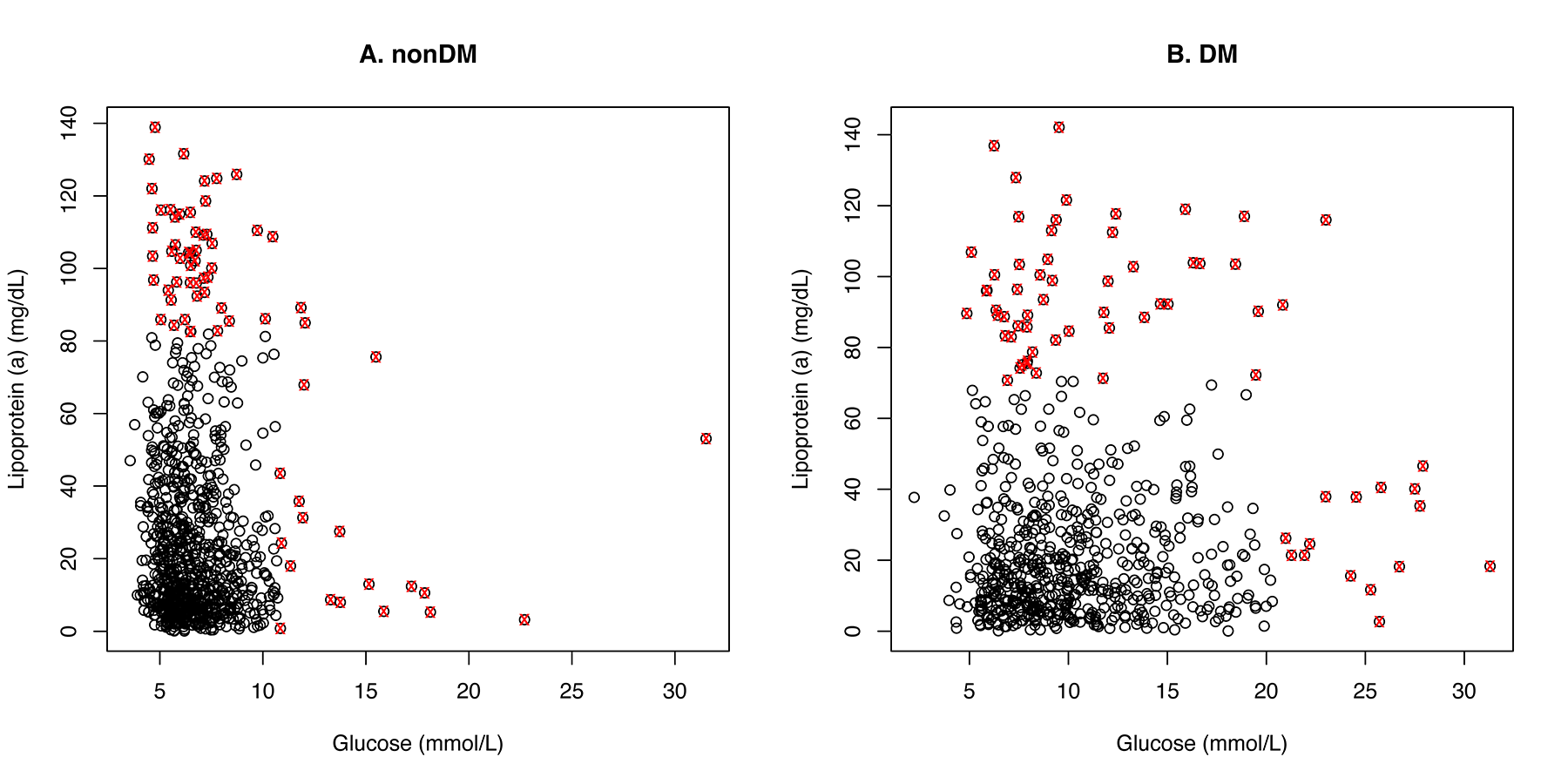

Supplement: Supplementary file 2 — Additional file 2: Figure S2. The scatter plot for the relationship between lipoproteinand glucose in patients with and without diabetes mellitus. Outliers are tested using a box plot test and marked in red, with 69in the nonDM group and 70in the DM group. [file 12933_2023_1881_MOESM2_ESM.tiff]

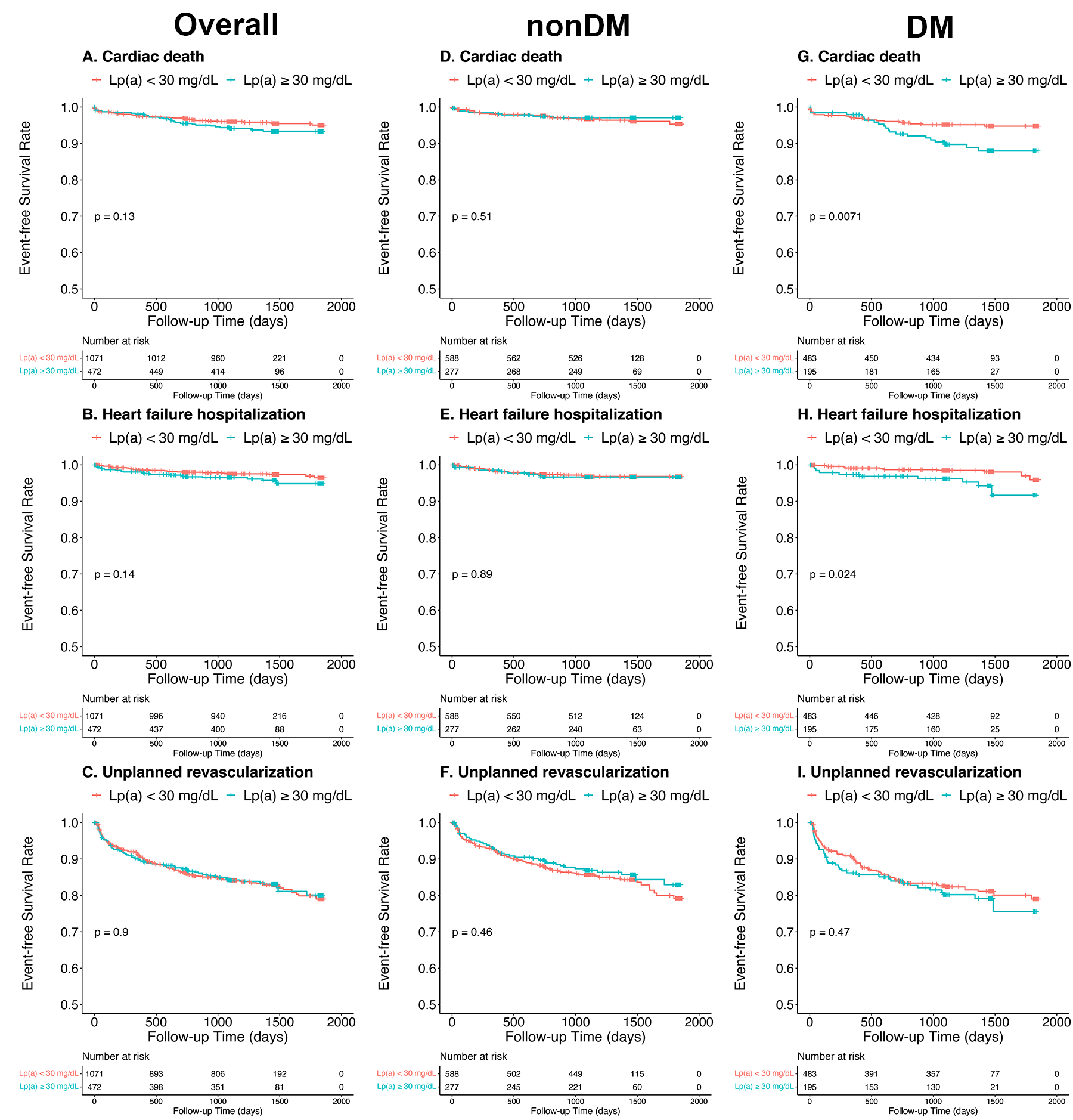

Supplement: Supplementary file 3 — Additional file 3: Figure S3. Kaplan–Meier curves for cumulative event-free survival rate between groups by lipoproteinlevels in overall, nonDM, and DM patients. DM, diabetes mellitus. [file 12933_2023_1881_MOESM3_ESM.tif]
