# Supplementary material for: Prognostic impacts of diabetes status and lipoprotein(a) levels in patients with ST-segment elevation myocardial infarction: a prospective cohort study
Source: Cardiovasc Diabetol. 2023 Jun 26;22:151. doi: 10.1186/s12933-023-01881-w (PMC10294355; doi:10.1186/s12933-023-01881-w)
Supplement: Supplementary file 15 — Additional file 15: Table S1. Baseline characteristics according to diabetes status. [file 12933_2023_1881_MOESM15_ESM.docx]

**Table S1** Baseline characteristics according to diabetes status.

|  | **Total (n = 1543)** | **nonDM (n = 865)** | **DM (n = 678)** | ***P*-value** |
| --- | --- | --- | --- | --- |
| Age (years) | 61.0 (52.0, 69.0) | 59.0 (50.5, 68.0) | 63.0 (54.0, 69.6) | < 0.001 |
| ≥ 65years | 210 (40.1) | 154 (39.1) | 56 (43.1) | 0.421 |
| Female | 295 (19.1) | 137 (15.8) | 158 (23.3) | < 0.001 |
| BMI (kg/m2) | 25.7 (23.4, 27.8) | 25.7 (23.4, 28.0) | 25.7 (23.4, 27.8) | 0.420 |
| Current Smoker | 1091 (70.7) | 638 (73.8) | 453 (66.8) | 0.003 |
| Hypertension | 997 (64.6) | 530 (61.3) | 467 (68.9) | 0.002 |
| Dyslipemia | 1387 (89.9) | 760 (87.9) | 627 (92.5) | 0.003 |
| Previous Stroke | 217 (14.1) | 94 (10.9) | 123 (18.1) | < 0.001 |
| CKD | 109 (7.1) | 58 (6.7) | 51 (7.5) | 0.534 |
| PAD | 82 (5.3) | 43 (5.0) | 39 (5.8) | 0.497 |
| Previous MI | 260 (16.9) | 127 (14.7) | 133 (19.6) | 0.010 |
| Previous PCI | 263 (17.0) | 121 (14.0) | 142 (20.9) | < 0.001 |
| GRACE score | 109.0 (90.0, 127.0) | 105.0 (85.0, 124.0) | 113.0 (94.0, 131.0) | < 0.001 |
| Killip (II- IV) | 209 (13.5) | 98 (11.3) | 111 (16.4) | 0.004 |
| LVEF (%) | 55.0 (50.0, 59.0) | 55.0 (50.0, 59.0) | 55.0 (48.0, 58.0) | 0.007 |
| LVEF < 50% | 383 (24.8) | 197 (22.8) | 186 (27.4) | 0.035 |
| MVD | 1150 (74.5) | 620 (71.7) | 530 (78.2) | 0.004 |
| eGFR(ml/min/1.732m^2^*) | 87.7 (72.9, 104.8) | 89.1 (74.5, 105.9) | 86.3 (70.7, 102.7) | 0.015 |
| Base cTnI (ng/mL) | 1.1 (0.1, 5.5) | 1.0 (0.1, 5.2) | 1.1 (0.1, 6.2) | 0.185 |
| Peak cTnI (ng/mL) | 16.0 (5.0, 38.4) | 16.0 (5.8, 37.3) | 16.1 (4.2, 38.5) | 0.470 |
| Base NT-proBNP (pg/mL) | 276.1 (68.5, 954.3) | 229.1 (58.8, 802.2) | 344.6 (84.8, 1104.0) | < 0.001 |
| Peak NT-proBNP (pg/mL) | 1459.0 (578.4, 3198.0) | 1385.0 (536.6, 3113.0) | 1622.0 (673.4, 3508.0) | 0.035 |
| TC (mmol/L) | 4.3 (3.6, 5.0) | 4.3 (3.6, 5.0) | 4.2 (3.6, 5.0) | 0.240 |
| Triglyceride (mmol/L) | 1.4 (1.0, 2.0) | 1.4 (0.9, 1.9) | 1.5 (1.1, 2.2) | < 0.001 |
| LDL-C (mmol/L) | 2.6 (2.0, 3.3) | 2.7 (2.1, 3.3) | 2.6 (2.0, 3.2) | 0.103 |
| HDL-C (mmol/L) | 1.0 (0.9, 1.2) | 1.1 (0.9, 1.3) | 1.0 (0.9, 1.2) | < 0.001 |
| Lipoprotein(a) (mg/dL) | 17.2 (7.6, 35.3) | 17.3 (7.6, 37.5) | 16.9 (7.8, 32.8) | 0.586 |
| Glucose (mmol/L) | 7.3 (6.0, 9.7) | 6.4 (5.5, 7.6) | 9.6 (7.4, 12.8) | < 0.001 |
| HbA1c (%) | 6.1 (5.7, 7.3) | 5.7 (5.5, 6.0) | 7.6 (6.8, 8.9) | < 0.001 |
| hsCRP (mg/L) | 6.0 (2.2, 10.9) | 5.5 (2.0, 10.6) | 6.6 (2.5, 11.1) | 0.017 |
| **Medication** |  |  |  |  |
| Baseline statins | 355 (23.0) | 184 (21.3) | 171 (25.2) | 0.067 |
| Follow-up statins | 829 (96.4) | 557 (95.5) | 272 (98.2) | 0.051 |
| Aspirin | 1474 (96.4) | 828 (96.3) | 646 (96.6) | 0.768 |
| Ticagrelor | 749 (49.0) | 438 (50.9) | 311 (46.5) | 0.085 |
| Clopidogrel | 777 (50.8) | 424 (49.3) | 353 (52.8) | 0.179 |
| ACEI/ARB | 1104 (72.2) | 623 (72.4) | 481 (71.9) | 0.814 |
| Βeta Blocker | 1330 (87.0) | 739 (85.9) | 591 (88.3) | 0.165 |
| Insulin | 155 (10.0) | - | 155 (22.9) | - |

Continuous variables are presented as medians (25−75th percentiles), and categorical variables are reported as counts (%). ACEI/ARB indicates angiotensin-converting enzyme inhibitors/angiotensin receptor blockers; BMI, body mass index; CKD, chronic kidney disease; cTnI, cardiac troponin I; DM, diabetes mellitus; GRACE, the Global Registry of Acute Coronary Events; HbA1c, hemoglobin A1c; HDL-C, high-density lipoprotein cholesterol; hsCRP, high-sensitivity C-reactive protein; LDL-C, low-density lipoprotein cholesterol; LVEF, left ventricular ejection fraction; MVD, multiple vessels disease; NT-proBNP, N-terminal pro B-type natriuretic peptide; PAD, peripheral artery disease; PCI, percutaneous coronary intervention; TC, total cholesterol.
