# Supplementary material for: Prognostic impacts of diabetes status and lipoprotein(a) levels in patients with ST-segment elevation myocardial infarction: a prospective cohort study
Source: Cardiovasc Diabetol. 2023 Jun 26;22:151. doi: 10.1186/s12933-023-01881-w (PMC10294355; doi:10.1186/s12933-023-01881-w)
Supplement: Supplementary file 16 — Additional file 16: Table S2. Association between lipoproteinlevels and risks of outcomes. [file 12933_2023_1881_MOESM16_ESM.docx]

Table S2 Association between lipoprotein(a) levels and risks of outcomes.

| **Outcome** | **Lp(a) (mg/dL)** | **Event (n/%)** | **Crude HR (95%CI)** | ***P*-value** | **Adjusted HR (95%CI)** * | ***P*-value** | ***P _for interaction_* ^†^** |
| --- | --- | --- | --- | --- | --- | --- | --- |
| **All-cause death** | **Overall patients** |  |  |  |  |  |  |
|  | Lp(a) ≥ 30 vs < 30 | 45 (9.5) | 1.10 (0.77~1.57) | 0.595 | 0.85 (0.59~1.23) | 0.389 | 0.025 |
|  | Lp(a) per SD | 137 (8.9) | 1.08 (0.92~1.26) | 0.335 | 1.01 (0.85~1.20) | 0.892 | 0.003 |
|  | Lp(a) < 10 | 45 (9.1) | 1 (Ref) |  | 1 (Ref) |  | 0.080 |
|  | 10 ≤ Lp(a) < 30 | 47 (8.1) | 0.92 (0.61~1.38) | 0.681 | 0.81 (0.53~1.23) | 0.326 |  |
|  | 30 ≤ Lp(a) < 50 | 24 (10.0) | 1.13 (0.69~1.86) | 0.623 | 0.74 (0.44~1.23) | 0.248 |  |
|  | Lp(a) ≥ 50 | 21 (9.0) | 0.98 (0.58~1.64) | 0.925 | 0.77 (0.45~1.33) | 0.356 |  |
|  | **Patients without DM** | |  |  |  |  |  |
|  | Lp(a) ≥ 30 vs < 30 | 15 (5.4) | 0.66 (0.37~1.19) | 0.167 | 0.56 (0.30~1.04) | 0.067 |  |
|  | Lp(a) per SD | 62 (7.2) | 0.82 (0.62~1.10) | 0.188 | 0.75 (0.56~1.01) | 0.056 |  |
|  | Lp(a) < 10 | 27 (9.6) | 1 (Ref) |  | 1 (Ref) |  |  |
|  | 10 ≤ Lp(a) < 30 | 20 (6.5) | 0.71 (0.40~1.26) | 0.236 | 0.71 (0.38~1.32) | 0.274 |  |
|  | 30 ≤ Lp(a) < 50 | 8 (5.9) | 0.61 (0.28~1.34) | 0.219 | 0.57 (0.24~1.32) | 0.187 |  |
|  | Lp(a) ≥ 50 | 7 (5.0) | 0.52 (0.23~1.19) | 0.121 | 0.40 (0.17~0.96) | 0.040 |  |
|  | **Patients with DM** | |  |  |  |  |  |
|  | Lp(a) ≥ 30 vs < 30 | 30 (15.4) | 1.68 (1.06~2.66) | 0.029 | 1.23 (0.75~2.02) | 0.419 |  |
|  | Lp(a) per SD | 75 (11.1) | 1.28 (1.06~1.54) | 0.009 | 1.32 (1.06~1.64) | 0.012 |  |
|  | Lp(a) < 10 | 18 (8.5) | 1 (Ref) |  | 1 (Ref) |  |  |
|  | 10 ≤ Lp(a) < 30 | 27 (10.0) | 1.19 (0.66~2.16) | 0.564 | 0.90 (0.48~1.68) | 0.736 |  |
|  | 30 ≤ Lp(a) < 50 | 16 (15.5) | 1.96 (1.00~3.86) | 0.050 | 0.82 (0.39~1.70) | 0.589 |  |
|  | Lp(a) ≥ 50 | 14 (15.2) | 1.74 (0.87~3.50) | 0.119 | 1.88 (0.89~3.99) | 0.100 |  |
| **reMI** | **Overall patients** |  |  |  |  |  |  |
|  | Lp(a) ≥ 30 vs < 30 | 24 (5.1) | 0.95 (0.59~1.54) | 0.842 | 0.82 (0.50~1.34) | 0.430 | 0.365 |
|  | Lp(a) per SD | 81 (5.2) | 1.02 (0.83~1.27) | 0.834 | 0.96 (0.77~1.19) | 0.680 | 0.403 |
|  | Lp(a) < 10 | 30 (6.1) | 1 (Ref) |  | 1 (Ref) |  | 0.187 |
|  | 10 ≤ Lp(a) < 30 | 27 (4.7) | 0.79 (0.47~1.33) | 0.369 | 0.78 (0.46~1.32) | 0.352 |  |
|  | 30 ≤ Lp(a) < 50 | 12 (5.0) | 0.85 (0.43~1.66) | 0.631 | 0.73 (0.37~1.46) | 0.381 |  |
|  | Lp(a) ≥ 50 | 12 (5.2) | 0.84 (0.43~1.64) | 0.613 | 0.70 (0.35~1.40) | 0.319 |  |
|  | **Patients without DM** | |  |  |  |  |  |
|  | Lp(a) ≥ 30 vs < 30 | 13 (4.7) | 0.75 (0.40~1.42) | 0.377 | 0.67 (0.35~1.30) | 0.238 |  |
|  | Lp(a) per SD | 49 (5.7) | 0.91 (0.67~1.22) | 0.525 | 0.87 (0.65~1.17) | 0.365 |  |
|  | Lp(a) < 10 | 17 (6.0) | 1 (Ref) |  | 1 (Ref) |  |  |
|  | 10 ≤ Lp(a) < 30 | 19 (6.2) | 1.05 (0.55~2.03) | 0.874 | 1.19 (0.60~2.35) | 0.613 |  |
|  | 30 ≤ Lp(a) < 50 | 8 (5.9) | 0.98 (0.42~2.27) | 0.960 | 0.98 (0.41~2.32) | 0.961 |  |
|  | Lp(a) ≥ 50 | 5 (3.5) | 0.58 (0.21~1.56) | 0.280 | 0.52 (0.19~1.47) | 0.219 |  |
|  | **Patients with DM** |  |  |  |  |  |  |
|  | Lp(a) ≥ 30 vs < 30 | 11 (5.6) | 1.39 (0.66~2.90) | 0.383 | 1.07 (0.48~2.38) | 0.876 |  |
|  | Lp(a) per SD | 32 (4.7) | 1.21 (0.89~1.64) | 0.223 | 1.11 (0.80~1.54) | 0.522 |  |
|  | Lp(a) < 10 | 13 (6.1) | 1 (Ref) |  | 1 (Ref) |  |  |
|  | 10 ≤ Lp(a) < 30 | 8 (3.0) | 0.50 (0.20~1.20) | 0.119 | 0.47 (0.18~1.17) | 0.106 |  |
|  | 30 ≤ Lp(a) < 50 | 4 (3.9) | 0.71 (0.23~2.18) | 0.546 | 0.44 (0.13~1.52) | 0.196 |  |
|  | Lp(a) ≥ 50 | 7 (7.6) | 1.30 (0.51~3.28) | 0.581 | 1.11 (0.40~3.05) | 0.842 |  |
| **Stroke** | **Overall patients** |  |  |  |  |  |  |
|  | Lp(a) ≥ 30 vs < 30 | 27 (5.7) | 1.21 (0.76~1.93) | 0.425 | 1.26 (0.78~2.03) | 0.337 | 0.009 |
|  | Lp(a) per SD | 78 (5.1) | 1.18 (0.97~1.43) | 0.098 | 1.20 (0.98~1.47) | 0.076 | 0.005 |
|  | Lp(a) < 10 | 21 (4.3) | 1 (Ref) |  | 1 (Ref) |  | 0.049 |
|  | 10 ≤ Lp(a) < 30 | 30 (5.2) | 1.27 (0.73~2.22) | 0.402 | 1.20 (0.68~2.12) | 0.530 |  |
|  | 30 ≤ Lp(a) < 50 | 11 (4.6) | 1.13 (0.54~2.34) | 0.748 | 1.17 (0.56~2.46) | 0.678 |  |
|  | Lp(a) ≥ 50 | 16 (6.9) | 1.64 (0.85~3.13) | 0.138 | 1.62 (0.84~3.16) | 0.153 |  |
|  | **Patients without DM** | |  |  |  |  |  |
|  | Lp(a) ≥ 30 vs < 30 | 6 (2.2) | 0.52 (0.21~1.26) | 0.148 | 0.52 (0.21~1.31) | 0.165 |  |
|  | Lp(a) per SD | 30 (3.5) | 0.77 (0.50~1.19) | 0.241 | 0.78 (0.50~1.23) | 0.283 |  |
|  | Lp(a) < 10 | 9 (3.2) | 1 (Ref) |  | 1 (Ref) |  |  |
|  | 10 ≤ Lp(a) < 30 | 15 (4.9) | 1.61 (0.71~3.69) | 0.258 | 1.78 (0.77~4.16) | 0.180 |  |
|  | 30 ≤ Lp(a) < 50 | 2 (1.5) | 0.46 (0.10~2.12) | 0.317 | 0.52 (0.11~2.44) | 0.406 |  |
|  | Lp(a) ≥ 50 | 4 (2.8) | 0.89 (0.27~2.89) | 0.848 | 0.90 (0.26~3.05) | 0.861 |  |
|  | **Patients with DM** |  |  |  |  |  |  |
|  | Lp(a) ≥ 30 vs < 30 | 21 (10.8) | 2.04 (1.16~3.62) | 0.014 | 2.04 (1.13~3.70) | 0.018 |  |
|  | Lp(a) per SD | 48 (7.1) | 1.43 (1.15~1.78) | 0.001 | 1.46 (1.16~1.84) | 0.001 |  |
|  | Lp(a) < 10 | 12 (5.7) | 1 (Ref) |  | 1 (Ref) |  |  |
|  | 10 ≤ Lp(a) < 30 | 15 (5.5) | 1.00 (0.47~2.14) | 0.999 | 0.96 (0.43~2.13) | 0.919 |  |
|  | 30 ≤ Lp(a) < 50 | 9 (8.7) | 1.73 (0.73~4.10) | 0.217 | 1.60 (0.65~3.93) | 0.304 |  |
|  | Lp(a) ≥ 50 | 12 (13.0) | 2.38 (1.07~5.29) | 0.034 | 2.47 (1.07~5.70) | 0.035 |  |
| **Cardiac death** | **Overall patients** |  |  |  |  |  |  |
|  | Lp(a) ≥ 30 vs < 30 | 29 (6.1) | 1.43 (0.90~2.27) | 0.134 | 1.10 (0.68~1.80) | 0.698 | 0.036 |
|  | Lp(a) per SD | 75 (4.9) | 1.25 (1.03~1.51) | 0.021 | 1.21 (0.98~1.50) | 0.074 | 0.013 |
|  | Lp(a) < 10 | 20 (4.0) | 1 (Ref) |  | 1 (Ref) |  | 0.047 |
|  | 10 ≤ Lp(a) < 30 | 26 (4.5) | 1.13 (0.63~2.03) | 0.672 | 1.00 (0.55~1.82) | 0.993 |  |
|  | 30 ≤ Lp(a) < 50 | 13 (5.4) | 1.37 (0.68~2.76) | 0.372 | 0.91 (0.44~1.90) | 0.811 |  |
|  | Lp(a) ≥ 50 | 16 (6.9) | 1.68 (0.87~3.24) | 0.121 | 1.33 (0.66~2.68) | 0.432 |  |
|  | **Patients without DM** | |  |  |  |  |  |
|  | Lp(a) ≥ 30 vs < 30 | 8 (2.9) | 0.76 (0.34~1.71) | 0.510 | 0.68 (0.28~1.64) | 0.392 |  |
|  | Lp(a) per SD | 30 (3.5) | 0.93 (0.64~1.35) | 0.708 | 0.88 (0.59~1.31) | 0.533 |  |
|  | Lp(a) < 10 | 13 (4.6) | 1 (Ref) |  | 1 (Ref) |  |  |
|  | 10 ≤ Lp(a) < 30 | 9 (2.9) | 0.66 (0.28~1.53) | 0.330 | 0.63 (0.25~1.63) | 0.345 |  |
|  | 30 ≤ Lp(a) < 50 | 3 (2.2) | 0.48 (0.14~1.69) | 0.252 | 0.57 (0.15~2.20) | 0.414 |  |
|  | Lp(a) ≥ 50 | 5 (3.5) | 0.77 (0.27~2.15) | 0.614 | 0.54 (0.17~1.70) | 0.292 |  |
|  | **Patients with DM** |  |  |  |  |  |  |
|  | Lp(a) ≥ 30 vs < 30 | 21 (10.8) | 2.19 (1.22~3.94) | 0.009 | 1.96 (1.00~3.84) | 0.050 |  |
|  | Lp(a) per SD | 45 (6.6) | 1.45 (1.17~1.81) | 0.001 | 1.68 (1.29~2.20) | < 0.001 |  |
|  | Lp(a) < 10 | 7 (3.3) | 1 (Ref) |  | 1 (Ref) |  |  |
|  | 10 ≤ Lp(a) < 30 | 17 (6.3) | 1.92 (0.79~4.62) | 0.147 | 1.64 (0.65~4.15) | 0.298 |  |
|  | 30 ≤ Lp(a) < 50 | 10 (9.7) | 3.08 (1.17~8.09) | 0.022 | 1.57 (0.54~4.58) | 0.405 |  |
|  | Lp(a) ≥ 50 | 11 (12.0) | 3.56 (1.38~9.19) | 0.009 | 5.60 (1.93~16.3) | 0.002 |  |
| **HF hospitalization** | **Overall patients** |  |  |  |  |  |  |
|  | Lp(a) ≥ 30 vs < 30 | 19 (4.0) | 1.55 (0.87~2.78) | 0.140 | 1.40 (0.77~2.56) | 0.274 | 0.116 |
|  | Lp(a) per SD | 47 (3.0) | 1.01 (0.77~1.34) | 0.920 | 0.97 (0.71~1.31) | 0.841 | 0.171 |
|  | Lp(a) < 10 | 12 (2.4) | 1 (Ref) |  | 1 (Ref) |  | 0.493 |
|  | 10 ≤ Lp(a) < 30 | 16 (2.8) | 1.19 (0.56~2.51) | 0.655 | 1.02 (0.47~2.20) | 0.958 |  |
|  | 30 ≤ Lp(a) < 50 | 13 (5.4) | 2.36 (1.08~5.17) | 0.032 | 1.85 (0.83~4.14) | 0.133 |  |
|  | Lp(a) ≥ 50 | 6 (2.6) | 1.06 (0.40~2.83) | 0.902 | 0.91 (0.33~2.55) | 0.863 |  |
|  | **Patients without DM** | |  |  |  |  |  |
|  | Lp(a) ≥ 30 vs < 30 | 9 (3.2) | 1.06 (0.48~2.36) | 0.889 | 0.82 (0.35~1.91) | 0.644 |  |
|  | Lp(a) per SD | 27 (3.1) | 0.84 (0.55~1.29) | 0.428 | 0.77 (0.47~1.24) | 0.277 |  |
|  | Lp(a) < 10 | 8 (2.8) | 1 (Ref) |  | 1 (Ref) |  |  |
|  | 10 ≤ Lp(a) < 30 | 10 (3.3) | 1.18 (0.46~2.98) | 0.730 | 1.43 (0.53~3.80) | 0.479 |  |
|  | 30 ≤ Lp(a) < 50 | 6 (4.4) | 1.59 (0.55~4.57) | 0.394 | 1.37 (0.45~4.13) | 0.579 |  |
|  | Lp(a) ≥ 50 | 3 (2.1) | 0.75 (0.20~2.82) | 0.670 | 0.60 (0.14~2.52) | 0.489 |  |
|  | **Patients with DM** |  |  |  |  |  |  |
|  | Lp(a) ≥ 30 vs < 30 | 10 (5.1) | 2.64 (1.10~6.35) | 0.030 | 3.66 (1.29~10.39) | 0.015 |  |
|  | Lp(a) per SD | 20 (2.9) | 1.24 (0.86~1.80) | 0.255 | 1.37 (0.88~2.15) | 0.166 |  |
|  | Lp(a) < 10 | 4 (1.9) | 1 (Ref) |  | 1 (Ref) |  |  |
|  | 10 ≤ Lp(a) < 30 | 6 (2.2) | 1.22 (0.34~4.32) | 0.760 | 1.01 (0.25~3.98) | 0.992 |  |
|  | 30 ≤ Lp(a) < 50 | 7 (6.8) | 4.33 (1.26~14.87) | 0.020 | 4.99 (1.21~20.57) | 0.026 |  |
|  | Lp(a) ≥ 50 | 3 (3.3) | 1.70 (0.38~7.62) | 0.486 | 2.11 (0.37~11.91) | 0.398 |  |
| **Unplanned revascularization** | **Overall patients** |  |  |  |  |  |  |
|  | Lp(a) ≥ 30 vs < 30 | 77 (16.3) | 0.98 (0.75~1.28) | 0.900 | 0.96 (0.73~1.26) | 0.753 | 0.466 |
|  | Lp(a) per SD | 255 (16.5) | 1.03 (0.91~1.16) | 0.665 | 1.00 (0.89~1.13) | 0.993 | 0.974 |
|  | Lp(a) < 10 | 71 (14.4) | 1 (Ref) |  | 1 (Ref) |  | 0.793 |
|  | 10 ≤ Lp(a) < 30 | 107 (18.5) | 1.36 (1.00~1.83) | 0.047 | 1.37 (1.01~1.86) | 0.042 |  |
|  | 30 ≤ Lp(a) < 50 | 38 (15.9) | 1.15 (0.78~1.71) | 0.483 | 1.19 (0.79~1.77) | 0.405 |  |
|  | Lp(a) ≥ 50 | 39 (16.7) | 1.18 (0.80~1.75) | 0.399 | 1.12 (0.75~1.66) | 0.591 |  |
|  | **Patients without DM** | |  |  |  |  |  |
|  | Lp(a) ≥ 30 vs < 30 | 39 (14.1) | 0.87 (0.60~1.26) | 0.461 | 0.89 (0.61~1.31) | 0.556 |  |
|  | Lp(a) per SD | 131 (15.1) | 1.01 (0.85~1.20) | 0.917 | 1.01 (0.85~1.20) | 0.906 |  |
|  | Lp(a) < 10 | 40 (14.2) | 1 (Ref) |  | 1 (Ref) |  |  |
|  | 10 ≤ Lp(a) < 30 | 52 (17.0) | 1.28 (0.85~1.93) | 0.241 | 1.25 (0.82~1.92) | 0.295 |  |
|  | 30 ≤ Lp(a) < 50 | 18 (13.2) | 0.92 (0.53~1.61) | 0.783 | 0.98 (0.56~1.73) | 0.948 |  |
|  | Lp(a) ≥ 50 | 21 (14.9) | 1.06 (0.62~1.79) | 0.842 | 1.04 (0.60~1.78) | 0.900 |  |
|  | **Patients with DM** |  |  |  |  |  |  |
|  | Lp(a) ≥ 30 vs < 30 | 38 (19.5) | 1.15 (0.79~1.69) | 0.469 | 1.09 (0.74~1.62) | 0.663 |  |
|  | Lp(a) per SD | 124 (18.3) | 1.05 (0.89~1.25) | 0.564 | 1.01 (0.84~1.20) | 0.942 |  |
|  | Lp(a) < 10 | 31 (14.6) | 1 (Ref) |  | 1 (Ref) |  |  |
|  | 10 ≤ Lp(a) < 30 | 55 (20.3) | 1.43 (0.92~2.22) | 0.111 | 1.55 (0.98~2.46) | 0.061 |  |
|  | 30 ≤ Lp(a) < 50 | 20 (19.4) | 1.46 (0.83~2.57) | 0.185 | 1.55 (0.87~2.76) | 0.140 |  |
|  | Lp(a) ≥ 50 | 18 (19.6) | 1.39 (0.78~2.48) | 0.269 | 1.30 (0.72~2.37) | 0.387 |  |

DM, diabetes mellitus; HF, heart failure; HR, hazard ratio; Lp(a), lipoprotein (a); reMI, recurrent myocardial infarction.

* Adjusted for age, sex, body mass index, hypertension, dyslipidemia, peripheral artery disease, chronic kidney disease, previous history of myocardial infarction and percutaneous coronary intervention, Killip class, the Global Registry of Acute Coronary Events risk score, multiple vessels disease, estimated glomerular filtration rate, left ventricular ejection fraction, and levels of total cholesterol, low-density lipoprotein cholesterol and high-sensitivity C-reactive protein, as well as the baseline and peak value of cardiac troponin I and N-terminal pro-B-type natriuretic peptide.

^†^*P _for interaction_* indicates the interaction of diabetes status on the relationship between Lp(a) and the risk of outcomes in the multivariable Cox regression.
