# Supplementary material for: Prognostic impacts of diabetes status and lipoprotein(a) levels in patients with ST-segment elevation myocardial infarction: a prospective cohort study
Source: Cardiovasc Diabetol. 2023 Jun 26;22:151. doi: 10.1186/s12933-023-01881-w (PMC10294355; doi:10.1186/s12933-023-01881-w)
Supplement: Supplementary file 19 — Additional file 19: Table S5. Association between lipoproteinlevels and risks of outcomes in patients without MACEs within 14 days or PCI-related complications. [file 12933_2023_1881_MOESM19_ESM.docx]

Table S5 Association between lipoprotein(a) levels and risks of outcomes in patients without MACEs within 14 days or PCI-related complications.

| **Outcome** | **Lp(a) (mg/dL)** | **Event (n/%)** | **Crude HR (95%CI)** | ***P*-value** | **Adjusted HR (95%CI)** * | ***P*-value** | ***P _for interaction_* ^†^** |
| --- | --- | --- | --- | --- | --- | --- | --- |
| **MACE** | **Overall patients** |  |  |  |  |  |  |
|  | Lp(a) ≥ 30 vs < 30 | 77 (16.6) | 1.03 (0.79~1.35) | 0.819 | 0.93 (0.70~1.23) | 0.615 | 0.004 |
|  | Lp(a) per SD | 247 (16.3) | 1.08 (0.96~1.22) | 0.198 | 1.04 (0.92~1.18) | 0.496 | 0.001 |
|  | Lp(a) < 10 | 79 (16.3) | 1 (Ref) |  | 1 (Ref) |  | 0.012 |
|  | 10 ≤ Lp(a) < 30 | 91 (16.1) | 1.01 (0.75~1.37) | 0.948 | 0.92 (0.68~1.25) | 0.586 |  |
|  | 30 ≤ Lp(a) < 50 | 38 (16.3) | 1.03 (0.70~1.51) | 0.887 | 0.86 (0.58~1.28) | 0.452 |  |
|  | Lp(a) ≥ 50 | 39 (17.0) | 1.05 (0.71~1.54) | 0.816 | 0.92 (0.62~1.36) | 0.671 |  |
|  | **Patients without DM** | |  |  |  |  |  |
|  | Lp(a) ≥ 30 vs < 30 | 30 (10.9) | 0.67 (0.44~1.01) | 0.058 | 0.62 (0.41~0.96) | 0.031 |  |
|  | Lp(a) per SD | 122 (14.3) | 0.86 (0.71~1.05) | 0.139 | 0.84 (0.69~1.03) | 0.088 |  |
|  | Lp(a) < 10 | 44 (15.8) | 1 (Ref) |  | 1 (Ref) |  |  |
|  | 10 ≤ Lp(a) < 30 | 48 (15.9) | 1.04 (0.69~1.57) | 0.848 | 1.10 (0.72~1.69) | 0.646 |  |
|  | 30 ≤ Lp(a) < 50 | 16 (11.9) | 0.75 (0.42~1.32) | 0.318 | 0.77 (0.43~1.40) | 0.390 |  |
|  | Lp(a) ≥ 50 | 14 (10.0) | 0.63 (0.34~1.14) | 0.126 | 0.56 (0.30~1.05) | 0.070 |  |
|  | **Patients with DM** |  |  |  |  |  |  |
|  | Lp(a) ≥ 30 vs < 30 | 47 (24.9) | 1.61 (1.12~2.31) | 0.010 | 1.49 (1.02~2.18) | 0.041 |  |
|  | Lp(a) per SD | 125 (18.9) | 1.31 (1.13~1.51) | < 0.001 | 1.35 (1.15~1.58) | < 0.001 |  |
|  | Lp(a) < 10 | 35 (16.8) | 1 (Ref) |  | 1 (Ref) |  |  |
|  | 10 ≤ Lp(a) < 30 | 43 (16.2) | 0.97 (0.62~1.52) | 0.905 | 0.79 (0.50~1.27) | 0.334 |  |
|  | 30 ≤ Lp(a) < 50 | 22 (22.2) | 1.46 (0.85~2.49) | 0.166 | 0.98 (0.56~1.71) | 0.938 |  |
|  | Lp(a) ≥ 50 | 25 (27.8) | 1.71 (1.02~2.87) | 0.040 | 1.84 (1.07~3.18) | 0.029 |  |
| **All-cause death** | **Overall patients** |  |  |  |  |  |  |
|  | Lp(a) ≥ 30 vs < 30 | 38 (8.2) | 1.10 (0.74~1.62) | 0.641 | 0.89 (0.59~1.33) | 0.561 | 0.023 |
|  | Lp(a) per SD | 116 (7.7) | 1.09 (0.92~1.29) | 0.330 | 1.01 (0.85~1.21) | 0.880 | 0.046 |
|  | Lp(a) < 10 | 39 (8.0) | 1 (Ref) |  | 1 (Ref) |  | 0.099 |
|  | 10 ≤ Lp(a) < 30 | 39 (6.9) | 0.88 (0.57~1.38) | 0.585 | 0.77 (0.48~1.21) | 0.252 |  |
|  | 30 ≤ Lp(a) < 50 | 20 (8.6) | 1.10 (0.64~1.88) | 0.731 | 0.76 (0.44~1.34) | 0.346 |  |
|  | Lp(a) ≥ 50 | 18 (7.8) | 0.96 (0.55~1.68) | 0.887 | 0.77 (0.43~1.37) | 0.374 |  |
|  | **Patients without DM** | |  |  |  |  |  |
|  | Lp(a) ≥ 30 vs < 30 | 13 (4.7) | 0.64 (0.34~1.19) | 0.159 | 0.52 (0.27~1.01) | 0.053 |  |
|  | Lp(a) per SD | 55 (6.4) | 0.81 (0.59~1.10) | 0.177 | 0.73 (0.53~0.99) | 0.046 |  |
|  | Lp(a) < 10 | 25 (9.0) | 1 (Ref) |  | 1 (Ref) |  |  |
|  | 10 ≤ Lp(a) < 30 | 17 (5.6) | 0.65 (0.35~1.20) | 0.170 | 0.68 (0.36~1.31) | 0.252 |  |
|  | 30 ≤ Lp(a) < 50 | 7 (5.2) | 0.58 (0.25~1.33) | 0.196 | 0.55 (0.22~1.34) | 0.186 |  |
|  | Lp(a) ≥ 50 | 6 (4.3) | 0.48 (0.20~1.16) | 0.103 | 0.35 (0.14~0.90) | 0.029 |  |
|  | **Patients with DM** | |  |  |  |  |  |
|  | Lp(a) ≥ 30 vs < 30 | 25 (13.2) | 1.76 (1.06~2.94) | 0.030 | 1.37 (0.79~2.37) | 0.259 |  |
|  | Lp(a) per SD | 61 (9.2) | 1.33 (1.08~1.62) | 0.006 | 1.34 (1.07~1.68) | 0.012 |  |
|  | Lp(a) < 10 | 14 (6.7) | 1 (Ref) |  | 1 (Ref) |  |  |
|  | 10 ≤ Lp(a) < 30 | 22 (8.3) | 1.26 (0.64~2.46) | 0.502 | 1.01 (0.50~2.05) | 0.982 |  |
|  | 30 ≤ Lp(a) < 50 | 13 (13.1) | 2.11 (0.99~4.50) | 0.052 | 1.04 (0.46~2.35) | 0.927 |  |
|  | Lp(a) ≥ 50 | 12 (13.3) | 1.92 (0.89~4.15) | 0.098 | 1.97 (0.86~4.50) | 0.109 |  |
| **reMI** | **Overall patients** |  |  |  |  |  |  |
|  | Lp(a) ≥ 30 vs < 30 | 23 (5.0) | 1.00 (0.61~1.64) | 0.987 | 0.86 (0.52~1.43) | 0.561 | 0.353 |
|  | Lp(a) per SD | 75 (5.0) | 1.06 (0.85~1.31) | 0.614 | 0.99 (0.79~1.23) | 0.904 | 0.482 |
|  | Lp(a) < 10 | 28 (5.8) | 1 (Ref) |  | 1 (Ref) |  | 0.155 |
|  | 10 ≤ Lp(a) < 30 | 24 (4.2) | 0.75 (0.44~1.30) | 0.305 | 0.73 (0.42~1.27) | 0.270 |  |
|  | 30 ≤ Lp(a) < 50 | 11 (4.7) | 0.84 (0.42~1.69) | 0.621 | 0.70 (0.34~1.45) | 0.338 |  |
|  | Lp(a) ≥ 50 | 12 (5.2) | 0.90 (0.46~1.78) | 0.769 | 0.76 (0.38~1.53) | 0.441 |  |
|  | **Patients without DM** | |  |  |  |  |  |
|  | Lp(a) ≥ 30 vs < 30 | 12 (4.4) | 0.78 (0.40~1.51) | 0.460 | 0.68 (0.34~1.35) | 0.266 |  |
|  | Lp(a) per SD | 44 (5.2) | 0.95 (0.70~1.29) | 0.746 | 0.90 (0.66~1.22) | 0.488 |  |
|  | Lp(a) < 10 | 15 (5.4) | 1 (Ref) |  | 1 (Ref) |  |  |
|  | 10 ≤ Lp(a) < 30 | 17 (5.6) | 1.07 (0.54~2.15) | 0.845 | 1.22 (0.59~2.51) | 0.592 |  |
|  | 30 ≤ Lp(a) < 50 | 7 (5.2) | 0.97 (0.40~2.38) | 0.949 | 0.98 (0.39~2.46) | 0.964 |  |
|  | Lp(a) ≥ 50 | 5 (3.6) | 0.65 (0.24~1.80) | 0.41 | 0.56 (0.19~1.60) | 0.278 |  |
|  | **Patients with DM** |  |  |  |  |  |  |
|  | Lp(a) ≥ 30 vs < 30 | 11 (5.8) | 1.47 (0.70~3.10) | 0.306 | 1.16 (0.51~2.61) | 0.727 |  |
|  | Lp(a) per SD | 31 (4.7) | 1.22 (0.90~1.66) | 0.202 | 1.15 (0.82~1.60) | 0.422 |  |
|  | Lp(a) < 10 | 13 (6.2) | 1 (Ref) |  | 1 (Ref) |  |  |
|  | 10 ≤ Lp(a) < 30 | 7 (2.6) | 0.43 (0.17~1.09) | 0.075 | 0.40 (0.15~1.04) | 0.061 |  |
|  | 30 ≤ Lp(a) < 50 | 4 (4.0) | 0.72 (0.23~2.21) | 0.563 | 0.43 (0.12~1.48) | 0.182 |  |
|  | Lp(a) ≥ 50 | 7 (7.8) | 1.31 (0.52~3.30) | 0.571 | 1.21 (0.44~3.34) | 0.718 |  |
| **Stroke** | **Overall patients** |  |  |  |  |  |  |
|  | Lp(a) ≥ 30 vs < 30 | 25 (5.4) | 1.12 (0.69~1.80) | 0.651 | 1.16 (0.71~1.89) | 0.544 | 0.018 |
|  | Lp(a) per SD | 76 (5.0) | 1.15 (0.94~1.41) | 0.169 | 1.17 (0.95~1.44) | 0.140 | 0.009 |
|  | Lp(a) < 10 | 21 (4.3) | 1 (Ref) |  | 1 (Ref) |  | 0.088 |
|  | 10 ≤ Lp(a) < 30 | 30 (5.3) | 1.27 (0.73~2.22) | 0.398 | 1.21 (0.69~2.13) | 0.512 |  |
|  | 30 ≤ Lp(a) < 50 | 10 (4.3) | 1.03 (0.48~2.18) | 0.948 | 1.07 (0.50~2.31) | 0.856 |  |
|  | Lp(a) ≥ 50 | 15 (6.5) | 1.53 (0.79~2.96) | 0.210 | 1.51 (0.77~2.97) | 0.235 |  |
|  | **Patients without DM** | |  |  |  |  |  |
|  | Lp(a) ≥ 30 vs < 30 | 6 (2.2) | 0.51 (0.21~1.26) | 0.146 | 0.52 (0.21~1.31) | 0.164 |  |
|  | Lp(a) per SD | 30 (3.5) | 0.77 (0.50~1.19) | 0.236 | 0.78 (0.50~1.23) | 0.282 |  |
|  | Lp(a) < 10 | 9 (3.2) | 1 (Ref) |  | 1 (Ref) |  |  |
|  | 10 ≤ Lp(a) < 30 | 15 (5.0) | 1.61 (0.71~3.68) | 0.258 | 1.79 (0.77~4.16) | 0.179 |  |
|  | 30 ≤ Lp(a) < 50 | 2 (1.5) | 0.46 (0.10~2.12) | 0.317 | 0.52 (0.11~2.44) | 0.406 |  |
|  | Lp(a) ≥ 50 | 4 (2.9) | 0.89 (0.27~2.87) | 0.839 | 0.90 (0.26~3.04) | 0.859 |  |
|  | **Patients with DM** |  |  |  |  |  |  |
|  | Lp(a) ≥ 30 vs < 30 | 19 (10.1) | 1.85 (1.03~3.33) | 0.040 | 1.83 (0.99~3.36) | 0.053 |  |
|  | Lp(a) per SD | 46 (6.9) | 1.40 (1.12~1.76) | 0.003 | 1.41 (1.11~1.79) | 0.005 |  |
|  | Lp(a) < 10 | 12 (5.8) | 1 (Ref) |  | 1 (Ref) |  |  |
|  | 10 ≤ Lp(a) < 30 | 15 (5.7) | 1.01 (0.47~2.15) | 0.989 | 0.97 (0.44~2.16) | 0.946 |  |
|  | 30 ≤ Lp(a) < 50 | 8 (8.1) | 1.54 (0.63~3.77) | 0.344 | 1.45 (0.58~3.67) | 0.430 |  |
|  | Lp(a) ≥ 50 | 11 (12.2) | 2.18 (0.96~4.94) | 0.062 | 2.20 (0.93~5.19) | 0.072 |  |
| **Cardiac death** | **Overall patients** |  |  |  |  |  |  |
|  | Lp(a) ≥ 30 vs < 30 | 25 (5.4) | 1.49 (0.90~2.47) | 0.123 | 1.21 (0.71~2.06) | 0.477 | 0.034 |
|  | Lp(a) per SD | 63 (4.2) | 1.29 (1.06~1.58) | 0.013 | 1.25 (1.000~1.55) | 0.048 | 0.011 |
|  | Lp(a) < 10 | 17 (3.5) | 1 (Ref) |  | 1 (Ref) |  | 0.050 |
|  | 10 ≤ Lp(a) < 30 | 21 (3.7) | 1.08 (0.57~2.05) | 0.806 | 1.00 (0.51~1.93) | 0.989 |  |
|  | 30 ≤ Lp(a) < 50 | 11 (4.7) | 1.38 (0.65~2.95) | 0.404 | 1.00 (0.45~2.22) | 0.999 |  |
|  | Lp(a) ≥ 50 | 14 (6.1) | 1.72 (0.85~3.5) | 0.131 | 1.45 (0.68~3.08) | 0.331 |  |
|  | **Patients without DM** | |  |  |  |  |  |
|  | Lp(a) ≥ 30 vs < 30 | 7 (2.6) | 0.73 (0.31~1.73) | 0.473 | 0.59 (0.23~1.50) | 0.266 |  |
|  | Lp(a) per SD | 27 (3.2) | 0.90 (0.60~1.35) | 0.612 | 0.82 (0.54~1.26) | 0.364 |  |
|  | Lp(a) < 10 | 12 (4.3) | 1 (Ref) |  | 1 (Ref) |  |  |
|  | 10 ≤ Lp(a) < 30 | 8 (2.7) | 0.63 (0.26~1.55) | 0.315 | 0.62 (0.23~1.64) | 0.334 |  |
|  | 30 ≤ Lp(a) < 50 | 3 (2.2) | 0.52 (0.15~1.84) | 0.312 | 0.58 (0.15~2.31) | 0.440 |  |
|  | Lp(a) ≥ 50 | 4 (2.9) | 0.66 (0.21~2.05) | 0.471 | 0.40 (0.11~1.43) | 0.160 |  |
|  | **Patients with DM** |  |  |  |  |  |  |
|  | Lp(a) ≥ 30 vs < 30 | 18 (9.5) | 2.53 (1.31~4.85) | 0.005 | 2.14 (1.03~4.43) | 0.041 |  |
|  | Lp(a) per SD | 36 (5.4) | 1.56 (1.24~1.97) | < 0.001 | 1.77 (1.33~2.34) | < 0.001 |  |
|  | Lp(a) < 10 | 5 (2.4) | 1 (Ref) |  | 1 (Ref) |  |  |
|  | 10 ≤ Lp(a) < 30 | 13 (4.9) | 2.07 (0.74~5.80) | 0.168 | 1.98 (0.68~5.78) | 0.211 |  |
|  | 30 ≤ Lp(a) < 50 | 8 (8.1) | 3.53 (1.15~10.8) | 0.027 | 1.84 (0.54~6.25) | 0.327 |  |
|  | Lp(a) ≥ 50 | 10 (11.1) | 4.53 (1.55~13.27) | 0.006 | 6.79 (2.08~22.22) | 0.002 |  |
| **HF hospitalization** | **Overall patients** |  |  |  |  |  |  |
|  | Lp(a) ≥ 30 vs < 30 | 19 (4.1) | 1.55 (0.87~2.78) | 0.139 | 1.41 (0.77~2.57) | 0.268 | 0.111 |
|  | Lp(a) per SD | 47 (3.1) | 1.01 (0.76~1.34) | 0.923 | 0.97 (0.72~1.32) | 0.857 | 0.161 |
|  | Lp(a) < 10 | 12 (2.5) | 1 (Ref) |  | 1 (Ref) |  | 0.484 |
|  | 10 ≤ Lp(a) < 30 | 16 (2.8) | 1.19 (0.56~2.51) | 0.653 | 1.02 (0.48~2.20) | 0.952 |  |
|  | 30 ≤ Lp(a) < 50 | 13 (5.6) | 2.37 (1.08~5.20) | 0.031 | 1.86 (0.83~4.15) | 0.131 |  |
|  | Lp(a) ≥ 50 | 6 (2.6) | 1.06 (0.40~2.83) | 0.905 | 0.92 (0.33~2.57) | 0.876 |  |
|  | **Patients without DM** | |  |  |  |  |  |
|  | Lp(a) ≥ 30 vs < 30 | 9 (3.3) | 1.05 (0.47~2.35) | 0.896 | 0.82 (0.35~1.91) | 0.640 |  |
|  | Lp(a) per SD | 27 (3.2) | 0.84 (0.55~1.29) | 0.420 | 0.76 (0.47~1.24) | 0.275 |  |
|  | Lp(a) < 10 | 8 (2.9) | 1 (Ref) |  | 1 (Ref) |  |  |
|  | 10 ≤ Lp(a) < 30 | 10 (3.3) | 1.18 (0.46~2.98) | 0.731 | 1.44 (0.54~3.84) | 0.469 |  |
|  | 30 ≤ Lp(a) < 50 | 6 (4.5) | 1.59 (0.55~4.57) | 0.393 | 1.37 (0.45~4.14) | 0.575 |  |
|  | Lp(a) ≥ 50 | 3 (2.1) | 0.74 (0.20~2.80) | 0.662 | 0.60 (0.14~2.53) | 0.490 |  |
|  | **Patients with DM** |  |  |  |  |  |  |
|  | Lp(a) ≥ 30 vs < 30 | 10 (5.3) | 2.66 (1.10~6.39) | 0.029 | 3.77 (1.32~10.73) | 0.013 |  |
|  | Lp(a) per SD | 20 (3.0) | 1.24 (0.86~1.81) | 0.251 | 1.43 (0.90~2.27) | 0.134 |  |
|  | Lp(a) < 10 | 4 (1.9) | 1 (Ref) |  | 1 (Ref) |  |  |
|  | 10 ≤ Lp(a) < 30 | 6 (2.3) | 1.22 (0.34~4.33) | 0.757 | 1.00 (0.25~3.97) | 0.995 |  |
|  | 30 ≤ Lp(a) < 50 | 7 (7.1) | 4.38 (1.28~15.04) | 0.019 | 4.94 (1.20~20.36) | 0.027 |  |
|  | Lp(a) ≥ 50 | 3 (3.3) | 1.71 (0.38~7.65) | 0.482 | 2.25 (0.39~12.98) | 0.364 |  |
| **Unplanned revascularization** | **Overall patients** |  |  |  |  |  |  |
|  | Lp(a) ≥ 30 vs < 30 | 76 (16.4) | 0.98 (0.75~1.29) | 0.899 | 0.96 (0.73~1.26) | 0.770 | 0.501 |
|  | Lp(a) per SD | 252 (16.6) | 1.03 (0.91~1.16) | 0.645 | 1.00 (0.89~1.13) | 0.954 | 0.833 |
|  | Lp(a) < 10 | 71 (14.6) | 1 (Ref) |  | 1 (Ref) |  | 0.973 |
|  | 10 ≤ Lp(a) < 30 | 105 (18.6) | 1.33 (0.98~1.80) | 0.063 | 1.34 (0.99~1.83) | 0.059 |  |
|  | 30 ≤ Lp(a) < 50 | 37 (15.9) | 1.13 (0.76~1.68) | 0.557 | 1.16 (0.78~1.74) | 0.467 |  |
|  | Lp(a) ≥ 50 | 39 (17) | 1.18 (0.80~1.75) | 0.403 | 1.12 (0.75~1.66) | 0.592 |  |
|  | **Patients without DM** | |  |  |  |  |  |
|  | Lp(a) ≥ 30 vs < 30 | 39 (14.2) | 0.88 (0.6~1.27) | 0.490 | 0.90 (0.61~1.32) | 0.591 |  |
|  | Lp(a) per SD | 130 (15.2) | 1.01 (0.85~1.20) | 0.896 | 1.01 (0.85~1.20) | 0.895 |  |
|  | Lp(a) < 10 | 40 (14.4) | 1 (Ref) |  | 1 (Ref) |  |  |
|  | 10 ≤ Lp(a) < 30 | 51 (16.9) | 1.25 (0.83~1.89) | 0.289 | 1.22 (0.80~1.87) | 0.360 |  |
|  | 30 ≤ Lp(a) < 50 | 18 (13.4) | 0.92 (0.53~1.61) | 0.782 | 0.98 (0.56~1.73) | 0.953 |  |
|  | Lp(a) ≥ 50 | 21 (15.0) | 1.05 (0.62~1.78) | 0.863 | 1.02 (0.59~1.76) | 0.935 |  |
|  | **Patients with DM** |  |  |  |  |  |  |
|  | Lp(a) ≥ 30 vs < 30 | 37 (19.6) | 1.14 (0.78~1.68) | 0.500 | 1.09 (0.73~1.62) | 0.682 |  |
|  | Lp(a) per SD | 122 (18.4) | 1.05 (0.89~1.25) | 0.553 | 1.01 (0.85~1.21) | 0.910 |  |
|  | Lp(a) < 10 | 31 (14.9) | 1 (Ref) |  | 1 (Ref) |  |  |
|  | 10 ≤ Lp(a) < 30 | 54 (20.4) | 1.41 (0.91~2.19) | 0.127 | 1.52 (0.96~2.41) | 0.076 |  |
|  | 30 ≤ Lp(a) < 50 | 19 (19.2) | 1.41 (0.79~2.49) | 0.243 | 1.48 (0.82~2.68) | 0.188 |  |
|  | Lp(a) ≥ 50 | 18 (20.0) | 1.40 (0.78~2.50) | 0.259 | 1.31 (0.72~2.39) | 0.375 |  |

DM, diabetes mellitus; HF, heart failure; HR, hazard ratio; Lp(a), lipoprotein (a); MACE, major adverse cardiovascular event (a composite of all-cause death, recurrent myocardial infarction, and stroke); reMI, recurrent myocardial infarction.

* Adjusted for age, sex, body mass index, hypertension, dyslipidemia, peripheral artery disease, chronic kidney disease, previous history of myocardial infarction and percutaneous coronary intervention, Killip class, the Global Registry of Acute Coronary Events risk score, multiple vessels disease, estimated glomerular filtration rate, left ventricular ejection fraction, and levels of total cholesterol, low-density lipoprotein cholesterol and high-sensitivity C-reactive protein, as well as the baseline and peak value of cardiac troponin I and N-terminal pro-B-type natriuretic peptide.

^†^*P _for interaction_* indicates the interaction of diabetes on the relationship between Lp(a) and the risk of outcomes in the multivariable Cox regression.
