# Supplementary material for: Prognostic impacts of diabetes status and lipoprotein(a) levels in patients with ST-segment elevation myocardial infarction: a prospective cohort study
Source: Cardiovasc Diabetol. 2023 Jun 26;22:151. doi: 10.1186/s12933-023-01881-w (PMC10294355; doi:10.1186/s12933-023-01881-w)
Supplement: Supplementary file 20 — Additional file 20: Table S6. Association between risks of outcomes and groups based on lipoproteinlevels and diabetes status in patients without MACEs within 14 days or PCI-related complications. [file 12933_2023_1881_MOESM20_ESM.docx]

**Table S6** Association between risks of outcomes and groups based on lipoprotein(a) levels and diabetes status in patients without MACEs within 14 days or PCI-related complications.

| **Outcome** | **Group** | **Event (n/%)** | **Crude HR (95%CI)** | ***P*-value** | **Adjusted HR (95%CI)** * | ***P*-value** |
| --- | --- | --- | --- | --- | --- | --- |
| MACE | nonDM, Lp(a) < 30 mg/dL | 92 (15.9) | 1 (Ref) |  | 1 (Ref) |  |
|  | nonDM, Lp(a) ≥ 30 mg/dL | 30 (10.9) | 0.67 (0.44~1.01) | 0.057 | 0.61 (0.40~0.93) | 0.022 |
|  | DM, Lp(a) < 30 mg/dL | 78 (16.5) | 1.06 (0.78~1.43) | 0.721 | 0.91 (0.67~1.24) | 0.550 |
|  | DM, Lp(a) ≥ 30 mg/dL | 47 (24.9) | 1.68 (1.18~2.39) | 0.004 | 1.26 (0.87~1.82) | 0.222 |
|  | nonDM, Lp(a) ≥ 30 mg/dL | 30 (10.9) | 1 (Ref) |  | 1 (Ref) |  |
|  | nonDM, Lp(a) < 30 mg/dL | 92 (15.9) | 1.49 (0.99~2.25) | 0.057 | 1.64 (1.08~2.51) | 0.022 |
|  | DM, Lp(a) < 30 mg/dL | 78 (16.5) | 1.58 (1.04~2.40) | 0.034 | 1.49 (0.97~2.30) | 0.068 |
|  | DM, Lp(a) ≥ 30 mg/dL | 47 (24.9) | 2.51 (1.59~3.97) | < 0.001 | 2.07 (1.29~3.31) | 0.003 |
| All-cause death | nonDM, Lp(a) < 30 mg/dL | 42 (7.3) | 1 (Ref) |  | 1 (Ref) |  |
|  | nonDM, Lp(a) ≥ 30 mg/dL | 13 (4.7) | 0.64 (0.34~1.19) | 0.159 | 0.52 (0.27~1.00) | 0.049 |
|  | DM, Lp(a) < 30 mg/dL | 36 (7.6) | 1.06 (0.68~1.66) | 0.791 | 0.85 (0.54~1.36) | 0.501 |
|  | DM, Lp(a) ≥ 30 mg/dL | 25 (13.2) | 1.87 (1.14~3.06) | 0.014 | 1.15 (0.68~1.95) | 0.594 |
|  | nonDM, Lp(a) ≥ 30 mg/dL | 13 (4.7) | 1 (Ref) |  | 1 (Ref) |  |
|  | nonDM, Lp(a) < 30 mg/dL | 42 (7.3) | 1.56 (0.84~2.91) | 0.159 | 1.91 (1.00~3.66) | 0.049 |
|  | DM, Lp(a) < 30 mg/dL | 36 (7.6) | 1.66 (0.88~3.13) | 0.117 | 1.63 (0.85~3.14) | 0.143 |
|  | DM, Lp(a) ≥ 30 mg/dL | 25 (13.2) | 2.92 (1.49~5.70) | 0.002 | 2.21 (1.10~4.43) | 0.026 |
| reMI | nonDM, Lp(a) < 30 mg/dL | 32 (5.5) | 1 (Ref) |  | 1 (Ref) |  |
|  | nonDM, Lp(a) ≥ 30 mg/dL | 12 (4.4) | 0.78 (0.40~1.51) | 0.458 | 0.69 (0.35~1.38) | 0.296 |
|  | DM, Lp(a) < 30 mg/dL | 20 (4.2) | 0.77 (0.44~1.35) | 0.362 | 0.68 (0.39~1.21) | 0.192 |
|  | DM, Lp(a) ≥ 30 mg/dL | 11 (5.8) | 1.09 (0.55~2.17) | 0.806 | 0.77 (0.37~1.58) | 0.471 |
|  | nonDM, Lp(a) ≥ 30 mg/dL | 12 (4.4) | 1 (Ref) |  | 1 (Ref) |  |
|  | nonDM, Lp(a) < 30 mg/dL | 32 (5.5) | 1.29 (0.66~2.50) | 0.458 | 1.44 (0.73~2.85) | 0.296 |
|  | DM, Lp(a) < 30 mg/dL | 20 (4.2) | 0.99 (0.48~2.03) | 0.981 | 0.98 (0.47~2.05) | 0.963 |
|  | DM, Lp(a) ≥ 30 mg/dL | 11 (5.8) | 1.40 (0.62~3.18) | 0.420 | 1.10 (0.47~2.57) | 0.820 |
| Stroke | nonDM, Lp(a) < 30 mg/dL | 24 (4.1) | 1 (Ref) |  | 1 (Ref) |  |
|  | nonDM, Lp(a) ≥ 30 mg/dL | 6 (2.2) | 0.51 (0.21~1.26) | 0.146 | 0.54 (0.22~1.34) | 0.183 |
|  | DM, Lp(a) < 30 mg/dL | 27 (5.7) | 1.43 (0.82~2.47) | 0.206 | 1.30 (0.74~2.29) | 0.359 |
|  | DM, Lp(a) ≥ 30 mg/dL | 19 (10.1) | 2.64 (1.45~4.82) | 0.002 | 2.45 (1.31~4.59) | 0.005 |
|  | nonDM, Lp(a) ≥ 30 mg/dL | 6 (2.2) | 1 (Ref) |  | 1 (Ref) |  |
|  | nonDM, Lp(a) < 30 mg/dL | 24 (4.1) | 1.94 (0.79~4.75) | 0.146 | 1.85 (0.75~4.56) | 0.183 |
|  | DM, Lp(a) < 30 mg/dL | 27 (5.7) | 2.77 (1.14~6.71) | 0.024 | 2.40 (0.98~5.89) | 0.055 |
|  | DM, Lp(a) ≥ 30 mg/dL | 19 (10.1) | 5.13 (2.05~12.86) | < 0.001 | 4.53 (1.78~11.52) | 0.001 |
| Cardiac death | nonDM, Lp(a) < 30 mg/dL | 20 (3.5) | 1 (Ref) |  | 1 (Ref) |  |
|  | nonDM, Lp(a) ≥ 30 mg/dL | 7 (2.6) | 0.73 (0.31~1.72) | 0.472 | 0.60 (0.24~1.48) | 0.265 |
|  | DM, Lp(a) < 30 mg/dL | 18 (3.8) | 1.11 (0.59~2.10) | 0.749 | 0.87 (0.44~1.69) | 0.672 |
|  | DM, Lp(a) ≥ 30 mg/dL | 18 (9.5) | 2.81 (1.49~5.31) | 0.001 | 1.71 (0.86~3.40) | 0.125 |
|  | nonDM, Lp(a) ≥ 30 mg/dL | 7 (2.6) | 1 (Ref) |  | 1 (Ref) |  |
|  | nonDM, Lp(a) < 30 mg/dL | 20 (3.5) | 1.37 (0.58~3.24) | 0.472 | 1.67 (0.68~4.13) | 0.265 |
|  | DM, Lp(a) < 30 mg/dL | 18 (3.8) | 1.52 (0.64~3.64) | 0.346 | 1.45 (0.59~3.56) | 0.420 |
|  | DM, Lp(a) ≥ 30 mg/dL | 18 (9.5) | 3.85 (1.61~9.23) | 0.002 | 2.86 (1.15~7.14) | 0.024 |
| HF hospitalization | nonDM, Lp(a) < 30 mg/dL | 18 (3.1) | 1 (Ref) |  | 1 (Ref) |  |
|  | nonDM, Lp(a) ≥ 30 mg/dL | 9 (3.3) | 1.05 (0.47~2.33) | 0.914 | 0.9 (0.4~2.05) | 0.807 |
|  | DM, Lp(a) < 30 mg/dL | 10 (2.1) | 0.68 (0.31~1.48) | 0.332 | 0.52 (0.23~1.15) | 0.107 |
|  | DM, Lp(a) ≥ 30 mg/dL | 10 (5.3) | 1.77 (0.81~3.83) | 0.150 | 1.26 (0.55~2.88) | 0.590 |
|  | nonDM, Lp(a) ≥ 30 mg/dL | 9 (3.3) | 1 (Ref) |  | 1 (Ref) |  |
|  | nonDM, Lp(a) < 30 mg/dL | 18 (3.1) | 0.96 (0.43~2.13) | 0.914 | 1.11 (0.49~2.52) | 0.807 |
|  | DM, Lp(a) < 30 mg/dL | 10 (2.1) | 0.65 (0.27~1.61) | 0.354 | 0.57 (0.23~1.45) | 0.238 |
|  | DM, Lp(a) ≥ 30 mg/dL | 10 (5.3) | 1.69 (0.69~4.16) | 0.254 | 1.39 (0.54~3.58) | 0.493 |
| Unplanned revascularization | nonDM, Lp(a) < 30 mg/dL | 91 (15.7) | 1 (Ref) |  | 1 (Ref) |  |
|  | nonDM, Lp(a) ≥ 30 mg/dL | 39 (14.2) | 0.88 (0.60~1.28) | 0.500 | 0.89 (0.61~1.30) | 0.546 |
|  | DM, Lp(a) < 30 mg/dL | 85 (18.0) | 1.18 (0.88~1.59) | 0.272 | 1.16 (0.86~1.57) | 0.325 |
|  | DM, Lp(a) ≥ 30 mg/dL | 37 (19.6) | 1.36 (0.93~1.99) | 0.117 | 1.25 (0.84~1.85) | 0.273 |
|  | nonDM, Lp(a) ≥ 30 mg/dL | 39 (14.2) | 1 (Ref) |  | 1 (Ref) |  |
|  | nonDM, Lp(a) < 30 mg/dL | 91 (15.7) | 1.14 (0.78~1.66) | 0.500 | 1.12 (0.77~1.64) | 0.546 |
|  | DM, Lp(a) < 30 mg/dL | 85 (18.0) | 1.34 (0.92~1.96) | 0.127 | 1.31 (0.89~1.92) | 0.171 |
|  | DM, Lp(a) ≥ 30 mg/dL | 37 (19.6) | 1.55 (0.99~2.42) | 0.058 | 1.40 (0.89~2.22) | 0.148 |

DM, diabetes mellitus; HF, heart failure; HR, hazard ratio; Lp(a), lipoprotein (a); MACE, major adverse cardiovascular event (a composite of all-cause death, recurrent myocardial infarction, and stroke); reMI, recurrent myocardial infarction.

* Adjusted for age, sex, body mass index, hypertension, dyslipidemia, peripheral artery disease, chronic kidney disease, previous history of myocardial infarction and percutaneous coronary intervention, Killip class, the Global Registry of Acute Coronary Events risk score, multiple vessels disease, estimated glomerular filtration rate, left ventricular ejection fraction, and levels of total cholesterol, low-density lipoprotein cholesterol and high-sensitivity C-reactive protein, as well as the baseline and peak value of cardiac troponin I and N-terminal pro-B-type natriuretic peptide.
