# Supplementary material for: Prognostic impacts of diabetes status and lipoprotein(a) levels in patients with ST-segment elevation myocardial infarction: a prospective cohort study
Source: Cardiovasc Diabetol. 2023 Jun 26;22:151. doi: 10.1186/s12933-023-01881-w (PMC10294355; doi:10.1186/s12933-023-01881-w)
Supplement: Supplementary file 21 — Additional file 21: Table S7. The details of restricted cubic spline fits for the relationships between continuous lipoproteinlevels and risks of outcomes in patients without MACEs within 14 days or PCI-related complications. [file 12933_2023_1881_MOESM21_ESM.docx]

**Table S7** The details of restricted cubic spline fits for the relationships between continuous lipoprotein(a) levels and risks of outcomes in patients without MACEs within 14 days or PCI-related complications.

|  | **MACE** | **All-cause death** | **reMI** | **Stroke** | **Cardiac death** | **Heart failure hospitalization** | **Unplanned revascularization** |
| --- | --- | --- | --- | --- | --- | --- | --- |
| **Overall patients** | | | | | | | |
| **Univariable Model** |  |  |  |  |  |  |  |
| C-index (*P*-value) | 0.504 (0.020) | 0.484 (0.029) | 0.549 (0.036) | 0.565 (0.035) | 0.570 (0.038) | 0.603 (0.045) | 0.537 (0.019) |
| Chi-square value | 2.34 | 1.74 | 2.61 | 2.19 | 6.97 | 5.98 | 3.21 |
| Degree of freedom | 3 | 3 | 3 | 3 | 3 | 3 | 3 |
| *P* for association | 0.505 | 0.627 | 0.456 | 0.537 | 0.072 | 0.113 | 0.361 |
| *P* for non-linearity | 0.765 | 0.731 | 0.309 | 0.850 | 0.865 | 0.067 | 0.222 |
| **­Multivariable Model** | |  |  |  |  |  |  |
| C-index (*P*-value) | 0.707 (0.017) | 0.802 (0.022) | 0.722 (0.028) | 0.709 (0.032) | 0.841 (0.028) | 0.821 (0.028) | 0.604 (0.018) |
| Overall chi-square value | 160.03 | 163.78 | 50.34 | 44.41 | 131.24 | 75.55 | 34.93 |
| Degree of freedom | 24 | 24 | 24 | 24 | 24 | 24 | 24 |
| Overall *P*-value | < 0.001 | < 0.001 | 0.001 | 0.007 | < 0.001 | < 0.001 | 0.069 |
| *P* for association | 0.541 | 0.499 | 0.549 | 0.499 | 0.139 | 0.162 | 0.436 |
| *P* for non-linearity | 0.444 | 0.314 | 0.352 | 0.900 | 0.570 | 0.081 | 0.256 |
| **Patients without DM** | | | | | | | |
| **Univariable Model** |  |  |  |  |  |  |  |
| C-index (*P*-value) | 0.538 (0.026) | 0.553 (0.039) | 0.516 (0.041) | 0.601 (0.051) | 0.548 (0.056) | 0.602 (0.045) | 0.532 (0.027) |
| Chi-square value | 3.11 | 2.74 | 0.19 | 3.83 | 0.97 | 2.92 | 2.30 |
| Degree of freedom | 3 | 3 | 3 | 3 | 3 | 3 | 3 |
| *P* for association | 0.375 | 0.433 | 0.979 | 0.280 | 0.810 | 0.404 | 0.513 |
| *P* for non-linearity | 0.637 | 0.674 | 0.959 | 0.285 | 0.704 | 0.240 | 0.322 |
| **­Multivariable Model** | |  |  |  |  |  |  |
| C-index (*P*-value) | 0.702 (0.025) | 0.809 (0.031) | 0.735 (0.040) | 0.702 (0.052) | 0.861 (0.044) | 0.842 (0.031) | 0.623 (0.025) |
| Overall chi-square value | 76.72 | 80.20 | 38.97 | 19.26 | 62.63 | 42.12 | 26.76 |
| Degree of freedom | 24 | 24 | 24 | 24 | 24 | 24 | 24 |
| Overall *P*-value | < 0.001 | < 0.001 | 0.028 | 0.738 | < 0.001 | 0.012 | 0.316 |
| *P* for association | 0.252 | 0.205 | 0.783 | 0.256 | 0.608 | 0.564 | 0.751 |
| *P* for non-linearity | 0.554 | 0.867 | 0.734 | 0.232 | 0.634 | 0.366 | 0.554 |
| **Patients with DM** | | | | | | | |
| **Univariable Model** |  |  |  |  |  |  |  |
| C-index (*P*-value) | 0.597 (0.027) | 0.588 (0.038) | 0.648 (0.056) | 0.641 (0.043) | 0.654 (0.046) | 0.639 (0.071) | 0.539 (0.026) |
| Chi-square value | 14.49 | 7.41 | 7.96 | 9.11 | 13.35 | 5.46 | 1.58 |
| Degree of freedom | 3 | 3 | 3 | 3 | 3 | 3 | 3 |
| *P* for association | 0.002 | 0.060 | 0.047 | 0.028 | 0.004 | 0.141 | 0.664 |
| *P* for non-linearity | 0.428 | 0.895 | 0.038 | 0.660 | 0.823 | 0.154 | 0.525 |
| **­Multivariable Model** | |  |  |  |  |  |  |
| C-index (*P*-value) | 0.757 (0.023) | 0.827 (0.029) | 0.834 (0.031) | 0.755 (0.039) | 0.877 (0.026) | 0.882 (0.036) | 0.611 (0.025) |
| Overall chi-square value | 135.52 | 102.14 | 46.02 | 47.52 | 80.49 | 43.32 | 18.97 |
| Degree of freedom | 24 | 24 | 24 | 24 | 24 | 24 | 24 |
| Overall *P*-value | < 0.001 | < 0.001 | 0.004 | 0.003 | < 0.001 | 0.009 | 0.754 |
| *P* for association | < 0.001 | 0.042 | 0.121 | 0.040 | 0.001 | 0.054 | 0.491 |
| *P* for non-linearity | 0.235 | 0.600 | 0.070 | 0.640 | 0.898 | 0.072 | 0.300 |

DM, diabetes mellitus; HR, hazard ratio; Lp(a), lipoprotein (a); MACE, major adverse cardiovascular event (a composite of all-cause death, recurrent myocardial infarction, and stroke); reMI, recurrent myocardial infarction.

* Adjusted for age, sex, body mass index, hypertension, dyslipidemia, peripheral artery disease, chronic kidney disease, previous history of myocardial infarction and percutaneous coronary intervention, Killip class, the Global Registry of Acute Coronary Events risk score, multiple vessels disease, estimated glomerular filtration rate, left ventricular ejection fraction, and levels of total cholesterol, low-density lipoprotein cholesterol and high-sensitivity C-reactive protein, as well as the baseline and peak value of cardiac troponin I and N-terminal pro-B-type natriuretic peptide.
